# Correction: Expanding syphilis test uptake using rapid dual self-testing for syphilis and HIV among men who have sex with men in China: A multiarm randomized controlled trial

**DOI:** 10.1371/journal.pmed.1004071

**Published:** 2022-08-04

**Authors:** Cheng Wang, Jason J. Ong, Peizhen Zhao, Ann Marie Weideman, Weiming Tang, M. Kumi Smith, Michael Marks, Hongyun Fu, Weibin Cheng, Fern Terris-Prestholt, Heping Zheng, Joseph D. Tucker, Bin Yang

In Table 4, the first two heading rows are not properly aligned. Please see the correct Table 4 below.

**Table 4:** HIV/STI testing and sexual behaviors self-reported by men who had syphilis testing during the trial and initiated at least 1 follow-up survey.

	SOC group	Standard SST group	Lottery incentivized SST group	Standard SST versus SOC		Lottery incentivized SST versus SOC		Lottery incentivized SST versus standard SST		
	Mean (SD)	Mean (SD)	Mean (SD)	MD (95% CI)^a^	*p*-Value	MD (95% CI)	*p*-Value	MD (95% CI)		*p*-Value
**Number of male sex partners in the past 3 months**										
Month 0 to 3	2.0 (1.0)	2.2 (1.7)	2.3 (1.9)	0.2 (−0.8, 1.1)	0.67	0.3 (−0.6, 1.3)	0.45	0.2 (−0.4, 0.8)	0.60	
Month 4 to 6	1.6 (1.0)	2.1 (1.7)	2.0 (1.6)	0.4 (−0.3, 1.1)	0.26	0.3 (−0.4, 1.0)	0.33	−0.1 (−0.7, 0.5)	0.78	
	***n*/*N* (%)**	***n*/*N* (%)**	***n*/*N* (%)**	**RD (95% CI)**	***p*-Value**	**RD (95% CI)**	***p*-Value**	**RD (95% CI)**	***p*-Value**	
**Male sex partners without condoms in the past 3 months**										
Month 0 to 3	2/7 (28.6)	22/66 (33.3)	26/68 (38.2)	4.8 (−37.1, 32.4)	0.98	9.7 (−32.8, 37.4)	0.81	4.9 (−11.7, 21.2)	0.60	
Month 4 to 6	5/13 (38.5)	20/45 (44.4)	24/62 (38.7)	6.0 (−26.2, 34.1)	0.82	0.3 (−31.5, 26.8)	1.00	−5.7 (−24.8, 13.5)	0.61	
**Anal group sex in the past 3 months**										
Month 0 to 3	2/7 (28.6)	5/74 (6.8)	6/72 (8.3)	−21.8 (−63.1, 3.2)	0.08	−20.2 (−61.3, 5.1)	0.10	1.6 (−7.9, 11.4)	0.77	
Month 4 to 6	1/14 (7.1)	5/51 (9.8)	9/69 (13.0)	2.7 (−24.9, 17.1)	0.84	5.9 (−22.1, 19.1)	0.70	3.2 (−9.8, 15.2)	0.61	
**Ever used substances before or during sex in the past 3 months**										
Month 0 to 3	3/7 (42.9)	31/74 (41.9)	40/72 (55.6)	−1.0 (−39.6, 32.7)	0.99	12.7 (−26.3, 46.2)	0.66	13.7 (−3.0, 29.7)	0.11	
Month 4 to 6	5/14 (35.7)	18/51 (35.3)	33/69 (47.8)	−0.4 (−31.0, 25.9)	1.00	12.1 (−19.0, 37.4)	0.47	12.5 (−5.8, 29.9)	0.17	
**Tested for HIV**										
Month 0 to 3	7/7 (100.0)	60/74 (81.8)	55/72 (76.4)	−18.9 (−31.3, 25.5)	0.26	−23.6 (−36.6, 20.4)	0.17	−4.7 (−18.3, 8.8)	0.53	
Month 4 to 6	14/14 (100.0)	48/51 (94.1)	62/69 (89.9)	−5.9 (−17.1, 18.6)	0.46	−10.1 (−20.6, 15.8)	0.24	−4.3 (−14.9, 7.5)	0.56	
Overall	19/20 (95.0)	83/90 (92.2)	80/90 (88.9)	−2.8 (−12.4, 18.3)	0.82	−6.1 (−16.5, 14.7)	0.50	−3.3 (−12.9, 5.7)	0.53	
**Tested for other STIs** ^b^										
Month 0 to 3	1/7 (14.3)	6/74 (8.1)	6/72 (8.3)	−6.2 (−49.1, 11.2)	0.79	−6.0 (−49.2, 11.5)	0.86	0.2 (−9.7, 10.1)	1.00	
Month 4 to 6	4/14 (28.6)	3/51 (5.9)	9/69 (13.0)	−22.7 (−51.7, −0.6)	0.02	−15.5 (−44.8, 6.6)	0.15	7.2 (−4.7, 18.4)	0.22	
Overall	8/20 (40.0)	14/90 (15.6)	20/90 (22.2)	−24.4 (−48.5, −0.9)	0.02	−17.8 (−42.5, 5.5)	0.10	6.7 (−5.2, 18.4)	0.28	

MD = mean difference. RD = risk (probability) difference expressed as a percentage.

aDue to rounding error, mean differences may differ by one decimal place from differences obtained by subtracting means listed in table.

bOther STIs included gonorrhea, chlamydia, human papillomavirus, and herpes simplex virus.

CI, confidence interval; SOC, standard of care; SST, syphilis self-testing; STI, sexually transmitted infection.
