# Supplementary material for: Expanding syphilis test uptake using rapid dual self-testing for syphilis and HIV among men who have sex with men in China: A multiarm randomized controlled trial
Source: PLoS Med. 2022 Mar 2;19(3):e1003930. doi: 10.1371/journal.pmed.1003930 (PMC8890628; doi:10.1371/journal.pmed.1003930)
Supplement: S1 Study Protocol — MSM, men who have sex with men; RCT, randomized controlled trial; SST, syphilis self-testing. (DOCX) [file pmed.1003930.s003.docx]

# Study protocol

**Promoting routine syphilis screening among men who have sex with men in China: study protocol for a randomised controlled trial of syphilis self-testing and lottery incentive**

**Abstract**

**Background:** Men who have sex with men (MSM) bear a high burden of syphilis infection. Expanding syphilis testing to improve timely diagnosis and treatment is critical to improve syphilis control. However, syphilis testing rates remain low among MSM, particularly in low- and middle-income countries. We describe the protocol for a randomised controlled trial (RCT) to assess whether provision of syphilis self-testing services can increase the uptake of syphilis testing among MSM in China.

**Methods:** 444 high-risk MSM will be recruited online and randomized in a 1:1:1 ratio to (1) standard syphilis self-testing arm; (2) a self-testing arm program enhanced with crowdsourcing and a lottery-based incentive, and (3) a standard of care (control). Self-testing services include a free syphilis self-test kit through the mail at monthly intervals. Participants in the lottery incentive arm will additionally receive health promotion materials generated from an open crowdsourcing contest and be given a lottery draw with a 10% chance to win 100 RMB (approximately 15 US Dollars) upon confirmed completion of syphilis testing. Syphilis self-test kits have step-by-step instructions and an instructional video. This is a non-blinded, open-label, parallel RCT. Participants in each arm will be followed-up at three and six months through WeChat (a social media app like Facebook messenger). Confirmation of syphilis self-test use will be determined by requiring participants to submit a photo of the used test kit to study staff via secure data messaging. Both self-testing and facility-based testing will be ascertained by sending a secure photographic image of the completed kit through an existing digital platform. The primary outcome is the proportion of participants who tested for syphilis in the past three months.

**Discussion:** Findings from this study will provide much needed insight on the impact of syphilis self-testing on promoting routine syphilis screening among MSM. The findings will also contribute to our understanding of the safety, effectiveness and acceptability of syphilis self-testing. These findings will have important implications for self-testing policy, both in China and internationally.

**Trial registration:** ChiCTR1900022409 (10 April, 2019)

**Background**

Men who have sex with men (MSM) are disproportionately affected by syphilis worldwide. The WHO estimates the prevalence of syphilis among MSM is 5% or more in at least 42 countries (15 countries in Asia including China with a prevalence of 5.1%), 10% or more in 20 countries ( 6 countries in Central America and 5 countries in South America ) and more than 20% in eight countries including 3 Pacific Island countries and 2 South American countries [[1](#_ENREF_1)]. Expanding access to syphilis testing so that individuals receive a timely diagnosis and treatment, plays a key role in the control of syphilis transmission [[2](#_ENREF_2)]. Evidence shows that for men who are HIV positive and/or MSM, screening every three months (compared with screening every six or twelve months) is associated with increased detection rates at various stages of syphilis infection [[3-7](#_ENREF_3)]. Increases in syphilis screening frequency are associated with increased detection of early asymptomatic syphilis with relative falls in secondary syphilis for both HIV-positive and HIV-negative MSM in Australia, suggesting a possible interruption of syphilis progression [[8](#_ENREF_8)].

However, given the cost of the tests, anticipated stigma, and the infrastructure requirements, syphilis testing rates are generally low, especially in resource-limited settings. Previous research shows that only 30% of Chinese MSM have ever tested for syphilis [[9](#_ENREF_9)]. Whilst traditional testing for syphilis is health facility-based, recent advances in testing techniques, including dried blood spots, point-of-care testing, and self-testing, have enabled decentralized testing strategies [[10](#_ENREF_10)]. Among these innovations, self-testing offers a great potential to expand syphilis testing among hard-to-reach populations due to its convenience, confidentiality, and relatively high sensitivity and specificity [[11](#_ENREF_11), [12](#_ENREF_12)]. Evidence from HIV self-testing studies have demonstrated its effectiveness in increasing HIV testing frequency among MSM in a variety of settings [[13-15](#_ENREF_13)]. In view of this, WHO subsequently released guidelines recommending HIV self-testing [[16](#_ENREF_16)].

Though not yet approved by the China Food and Drug Administration, syphilis self-test kits are currently available for purchase in China, mainly through online retailers [[17](#_ENREF_17)]. In 2018, we conducted a nationwide Internet survey to explore the prevalence of and correlates of syphilis self-testing uptake among MSM in China. Results showed that half of MSM who ever tested for syphilis had ever used a self-test. Self-testing potentially reached MSM who would not otherwise have attended health facilities for testing [[18](#_ENREF_18)]. Several studies in literature have explored the use of syphilis self-testing among MSM [[12](#_ENREF_12), [19-21](#_ENREF_19)], few studies have examined the effectiveness of intervention strategies which found that Internet-based strategy, such as using Facebook or smartphone applications, could be an effective way to promote syphilis self-testing uptake. Further evidence to inform the design of interventions by using self-testing to promote routine syphilis screening and linkages to care and treatment among MSM is needed.

Economic incentives (cash or in-kind rewards) to promote healthy behaviors are becoming increasingly common and have been suggested as an effective approach to promote healthy behaviors [[22](#_ENREF_22)]. We found Chinese MSM who had higher risk attitudes and who reported riskier sexual behaviours indicated greater interest in taking a syphilis testing for the opportunity to participate in a lottery draw [[23](#_ENREF_23)]. Thus, lottery incentive may be useful in promoting syphilis screening among high risk MSM. However, the mode of the lottery and the amount of incentives are unknown. Crowdsourcing organizes a group of non-experts and experts to solve a problem, which presents a promising strategy in creating effective intervention for the target populations [[24](#_ENREF_24)]. This approach has demonstrated effectiveness in increasing HIV testing by 43% among MSM and spurred community engagement [[25](#_ENREF_25)]. Thus, to enhance the syphilis self-testing with lottery incentive by using crowdsourcing may be a promising way for service delivery.

The purpose of this protocol manuscript is to describe a randomized controlled trial that will determine the effectiveness of two syphilis self-testing strategies compared to conventional facility-based syphilis testing.

**Methods/design**

**Study objectives**

This randomized controlled trial (RCT) is designed to determine whether provision of syphilis self-testing increases the proportion of participants who tested for syphilis during the trial among MSM in China. In addition, this study also aims to explore whether integration of a crowdsourced lottery incentive can further enhance the promotion of syphilis testing and linkage to care, after testing positive, among MSM in China.

**Study design**

This is a non-blinded, open-label, parallel RCT with individuals randomized in a 1:1:1 ratio to three study arms: control arm (standard of care); standard syphilis self-testing arm; and lottery incentivized syphilis self-testing arm. Control arm participants will receive information on self-referral pathways for free facility-based syphilis testing. Both self-testing arms will be offered to access syphilis self-test kits for free at monthly intervals. Participants in each arm will be followed-up for six months (Figure 1). The study hypothesis had been tested in a pilot study, completed in October 2019, which examined the feasibility of implementing the self-testing and crowdsourced lottery incentivized syphilis self-testing model among 145 Chinese MSM. The pilot study showed that both standard self-testing arm (0.74) and lottery incentivized arm (0.70) increased the frequency of syphilis testing more than the control arm (0.36). Preliminary data from this pilot study was used to inform this definitive trial design. Preliminary results are available on the supplement materials. The definitive trial, outlined in the current trial protocol, has begun in December 2019, and is expected to be completed in July 2020.

**Study setting and population**

Participants will be recruited from a large MSM-oriented mobile social app - Blued (Danlan, Beijing, China). Blued is China's most popular social networking mobile application among MSM. By February 2018, Blued had 40 million registered users globally, with 70% in Mainland China [[26](#_ENREF_26)].

Participants are eligible if they: are born biologically male; aged 18 years or above; had unprotected anal sex with other men in the past twelve months; reported not testing for syphilis in the last six months; not currently living with syphilis; plan to live in China for the next six months; and have a stable residence where they can securely receive a postal package. Participants are excluded if they: are participating in another research programme that is related to HIV/STIs or cannot provide consent.

**Arms and Interventions**

Arms and interventions are summarized in Table 1.

**1. Control arm:** Participants in the control arm will be provided information about local STD services and encouraged to uptake free syphilis testing at their local STD or HIV voluntary testing and counseling (HTC) clinics. HTC clinics in China provide both HIV and syphilis testing free of charge. Participants will be encouraged to screen for syphilis every three months. Reminder will be sent by SMS or WeChat (a social media app like Twitter and Facebook, commonly used in China) three monthly (enrollment, three-month, and six-month) during six months study period.

**2. Standard self-testing arm**: In addition to being prompted to uptake syphilis testing at local facilities, participants in this arm will be provided with access to syphilis self-testing along with the reminders. Participants may request order of syphilis self-testing packages through WeChat with the package being sent to participants by postal mail (express delivery). The provision of syphilis self-testing service will last for six months. Participants order self-testing package online with maximum one self-testing package per month.

**3. Lottery incentivized self-testing arm**: In this arm, the participant will be provided with both control arm and standard syphilis self-testing arm interventions. In addition, participants who confirm that they have tested for syphilis during the trial period either through self-testing or facility-testing will be given a lottery draw. The lottery draw will provide a 10% chance of winning 100 RMB (equal to 15 US Dollars). The procedure, value and probability of the lottery scheme was determined by a previous open crowdsourcing contest. We solicited 14 lottery schemes via an open crowdsourcing contest. The winning entries were then used to develop the aforementioned lottery incentivizes to be used in the current study.

**Table 1 Arms and interventions information**

| **Arms** | **Intervention description** | **Intervention delivery** |
| --- | --- | --- |
| **Control arm** | 1. **Test prompts**: information about risk of syphilis infection and local free syphilis test services. | - Text messages will be sent at enrollment, three-month visit, and six-month visit via WeChat. - Voluntary report testing result to the platform. |
| **Standard self-testing arm** | 1. **Test prompts**: information about risk of syphilis infection and local free syphilis test services.  2. **Self-testing**: free home delivery of self-testing package. | - Text messages will be sent at enrollment, three-month visit, and six-month visit via WeChat. - Provision of syphilis self-testing service: self-testing package can be ordered online and sent to participant through postal mail. Maximum one self-testing package per month. - Voluntary report self-test result to the platform. |
| **Lottery incentivized self-testing arm** | 1. **Test prompts**: information about risk of syphilis infection and local free syphilis test services.  2. **Self-testing**: free home delivery of self-testing package.  3. **Lottery incentive**: lottery draw with 10% chance to win 100 RMB for participants have confirmed uptake of syphilis testing. | - Text messages will be sent at enrollment, three-month visit, and six-month visit via WeChat. - Provision of syphilis self-testing service: self-testing package can be ordered online and sent to participant through postal mail. Maximum one self-testing package per month. - Voluntary report self-test result to the platform. - Lottery draw will be given to participants who get syphilis tested and upload photo verification to the platform. |

***Syphilis self-testing kit***

The HIV self-testing practice in China, using rapid test kits for self-testing, has shown good acceptability, feasibility, and safety [[11](#_ENREF_11), [12](#_ENREF_12), [27](#_ENREF_27)]. In this trial, we will use the syphilis and HIV combo rapid test kit (SD BIOLINE Syphilis/HIV Duo). The SD BIOLINE Syphilis/HIV Duo test is a solid phase immunochromatographic assay for the qualitative detection of antibodies to all isotypes (IgG, IgM, and IgA) specific to HIV-1/2 and/or Treponema pallidum (TP) simultaneously in human serum, plasma, or whole blood. The sensitivity and specificity for syphilis is 99.67% and 99.72% respectively [[28](#_ENREF_28)]. Our pilot trial results showed that the overall satisfaction with the use of syphilis self-testing was 100%. The syphilis self-testing package will be equipped with the manufacturer-supplied step-by-step instructions and a web-link (Source link: https://www.alere.com/en/home/support/product-demos/sd-bioline-hiv-syphilis-duo.html) to an instructional video.

**Outcomes Measures**

The primary outcome is the proportion of participants who tested for syphilis during the trial. Syphilis tests may be either at a clinic or via self-test. This will be verified in all participants using photo-verification. We will additionally look at the incremental benefit of the lottery incentive efficacy in promoting syphilis, number of newly identified syphilis infections, the linkage to syphilis clinical care after self-testing, the proportion of HIV and other STIs (chlamydia, gonorrhea) tests among the participants who use self-testing and cost-effectiveness of syphilis self-testing in promoting uptake of syphilis testing among Chinese MSM.

**Study procedures**

***Recruitment***

A banner link will be posted on the Blued’s portal website and startup screen. Recruitment announcements of the study will also be sent via other social media portals (WeChat; Weibo, a microblogging platform; and QQ, a messaging platform).

***Randomisation and allocation***

We will use block randomization with a block size of 12. Computer-generated randomisation codes will be produced and kept by a biostatistician who is not involved in participant enrollment.

Participant enrollment will be carried out online by a research assistant. Interested participants give consent and complete eligibility screening. Eligible participants then complete a baseline survey. Participants who complete the baseline survey are directed to contact the research assistant who is responsible for allocating the participants into one of the three intervention arms based on the randomization code. Participants will be provided with 20 RMB (equal to 3 US Dollars) for their time to complete the baseline survey.

***Blinding***

As it would not be possible to blind participants to their study arms, this is a single blinded study. Investigators and staff assessing the outcomes will be blinded to participants' group assignments.

***Follow-up support***

A research assistant will be responsible for participants’ retention management and care support through WeChat and telephone (office hours: Monday to Friday from 8:00 am to 5:30 pm). Care support includes pre-test counselling, instruction of use of self-test kit, interpretation of results, advice for reactive test results, and other related emergency events. A log of each enquiry will be recorded.

***Follow-up on reactive self-tests***

Participants are advised to inform the research assistant if they have any reactive self-test results. Any participant with a reactive self-test result will be referred to undergo confirmatory laboratory testing and clinical examination at the designated clinics/ hospitals. The laboratory confirmation and management of participants with syphilis will be based on the standard protocols at the respective clinic. If the participants are diagnosed with syphilis, we will undertake further follow-up to obtain treatment information.

***Data collection***

**Syphilis testing record and results:** Confirmed syphilis self-testing will be determined by photo verification of the used test kit. Facility-based syphilis testing and result will be determined by photo verification of the test report. Participants in all three arms who upload photo verification will get 5 RMB (approximately equal to 0.7 US dollars).

**Surveys**

**Baseline:** All participants complete a self-administered survey on an online survey platform (Wen Juan Xing, https://www.wjx.cn) at enrolment. The baseline survey includes questions on socio-demographic characteristics, sexual behaviors, past HIV/syphilis testing and treatment, and attitude towards syphilis self-testing.

**Follow-up surveys:** Participants in the three arms of the study will complete a brief online survey at three-monthly intervals. The follow up surveys collects information on the number, time, location, reasons for, and results of syphilis tests (including self-tests) and other STI tests at clinics and community sites, and sexual risk behaviors in the past three months. Participants in the two self-testing arms are also asked about the number of self-test kits they used to test their sexual partner(s) or if they gave any kits to someone else to test. At the last follow-up, participants will be asked to get STD (HIV, syphilis, chlamydia, and gonorrhea) screening test in their nearby clinic. The testing fee will be redeemed by an upload of the test results. Participants will be provided with 30 RMB (equal to 4 US Dollars) for their time to complete each follow-up survey.

**Statistical methods**

***Sample size***

According to our initial pilot study (***Supplementary material***), the proportion of individuals who undertook a syphilis test was 0.36 (95% confidence interval: 0.22 – 0.52) at the one-month follow-up in the control arm. This increased to 0.74 (95% confidence interval: 0.61 – 0.81) in the self-testing arm. On this basis, for the primary outcome with 80% statistical power and type I error probability of 0.05, we conservatively estimate a sample size of 29 per arm (including 20% loss to follow up [25]) for this study. According to our previous experience on using the crowdsourced HIV self-testing intervention in increasing HIV testing among MSM (the proportion was 0.85 [25]), we estimate a sample size of 148 for each arm to achieve 80% power to detect a difference between the crowdsourced lottery incentivized self-testing arm and standard syphilis self-testing arm. Finally, a total of 444 (148 per arm) will be enrolled in this trial.

#### *Analysis plan*

Data will be retrieved from the online survey platform. De-identified datasets and codebook will be stored for statistical analysis. The syphilis testing proportion in each arm (control, standard self-testing, and lottery incentivized self-testing) will be assessed using an intention-to-treat analysis during the follow-up period, excluding confirmatory tests after a reactive self-test.

The effects of the intervention will be measured by comparing the differences in proportions of men who take syphilis testing during two rounds of follow up by using generalized estimating equation (GEE) model. We will use the chi-square test to compare differences in the proportion of linkage to syphilis clinical care, have HIV and other STI (chlamydia, gonorrhea) testing performed, and have condomless sex events with different partners within six months of randomization across three groups and use post-host test to check the significance of differences between each of the two groups.

We will estimate the total and incremental unit costs for each arm. All costs will be identified as start-up costs, fixed costs and variable costs. Start-up costs are incurred during the process of creating this study (e.g. survey platform coordination). Fixed costs are costs that do not change with an increase or decrease in the number of people served during the trial period (e.g. administration, office equipment). Variable costs are expenses that vary with the number of participants served (e.g. recruitment, supplies, postage fee). We will first calculate the total cost for each group, then divided these costs by the number of men tested and the number of newly identified syphilis cases detected in each study arm to obtain the incremental unit costs. In addition, we will build a decision tree model using a healthcare provider perspective to estimate the incremental cost for per additional person tested and case identified for each intervention will be calculated. Univariate, multivariate and probabilistic sensitivity analyses will be conducted to test the robustness of our results.

***Missing data plan***

We anticipate the loss to follow-up will be less than 20% for the first three months. If outcome measures are missing less than 11% of participants, then the primary analysis will use a complete-case approach. If the outcome measures are missing for 11% to <20% of participants, then a sensitivity analysis using multiple imputation will be used to compute the missing outcome of the three arms.

***Ethical considerations***

IRB approval was obtained from the Dermatology Hospital of Southern Medical University (GDDHLS-20181206). All participants will be provided an online consent form prior to study initiation. This online informed consent describes what personal data will be collected, and explains that data will be used for research purposes only. Contact information will be provided for participants for further enquiry. Participants will be required to ‘click-to-consent’ and verified through mobile telephone as agreement to proceed with the study.

***Trial registration***

The study has been registered with the Chinese Clinical Trial Registry (trial ID [ChiCTR1900022409](http://www.chictr.org.cn/showproj.aspx?proj=37582)).

**Discussion**

In this study, we will test three syphilis testing models (standard of care, syphilis self-testing, and crowdsourced lottery incentivized self-testing) in a parallel randomized controlled trial. With the successful experience of HIV self-testing, we hypothesize that syphilis self-testing could increase syphilis test uptake by overcoming the structural barriers that are innate within traditional facility testing services. Findings from this study will contribute to the scarce data on the impact of syphilis self-testing on promoting routine syphilis screening among MSM. The findings will also contribute to our understanding of the safety, effectiveness and acceptability of syphilis self-testing. These findings will have important implications for self-testing policy, both in China and internationally.

Several implementation challenges that have emerged from HIV self-testing practice have limited its public health impact. Many are concerned that self-testing does not sufficiently connect users with critical post-testing services, such as confirmatory testing and management of those who test positive and that these limitations may result in delayed linkage to care [[11](#_ENREF_11), [27](#_ENREF_27), [29](#_ENREF_29), [30](#_ENREF_30)]. For syphilis self-testing, this problem could be more salient. The majority of syphilis rapid diagnostic tests detect treponemal specific antibodies. Though this has high sensitivity, it cannot distinguish past from current infections. A solution to encourage those whose self-test is reactive to undertake laboratory confirmation at the local clinic therefore needs to be developed. We have therefore introduced a new model of crowdsourced lottery incentivized syphilis self-testing, which integrates a behavioral economics tool of conditional monetary incentives enhanced by a crowdsourced approach, with the aim of enhancing syphilis testing uptake and linkage to care [[31](#_ENREF_31)]. However, the lottery prize amount, and how frequently incentives should be offered varies by populations and countries. We adopted a crowdsourcing method by organizing an opening contest from the target community to inform design an optimized financial incentive model in helping delivery of syphilis testing service (include self-testing) and linkage to care [[25](#_ENREF_25), [32](#_ENREF_32), [33](#_ENREF_33)]. This is a major innovation of this study and, if successful, it will provide insights to inform design of optimal self-testing model by using crowdsourcing method and tools of behavioral economics.

The major challenge of this study is to retrieve validated testing proof to verify self-testing results. Our previous study reported that 47% of men submitted proof of syphilis self-testing with 20 RMB (about 3 US dollar) cash incentive [[18](#_ENREF_18)]. This was a cross sectional survey, and people are not always able to find the required proof, especially for those who tested a long time ago. Furthermore, we cannot guarantee that submitted photographic proofs truly belongs to the patient. We previously found that more than 30% of men would submit a proof of testing even if no incentive was offered in an HIV self-testing study [[25](#_ENREF_25)]. To increase the retrieval rate, we will provide a small cash incentive (5 RMB) to reimburse their submission of self-testing results or other testing proof materials. To link self-test kits with the user, we will code each self-testing package and direct them to take a photo of the used strip with the background of the package, which has been successfully tested in our pilot trial.

Another potential limitation of this study is the possibility of contamination between arms. We cannot prevent participants from the control arm to purchase syphilis self-testing kits for use through online e-market which we have been previously found in some MSM in China [[18](#_ENREF_18)]. To mitigate this, we will record the participants’ testing method in each group and if necessary adjust for this in the data analysis. The six-month duration of follow-up also limits the ability of the study to assess the long-term effect of the intervention on syphilis incidence.

Findings from this proposed study will be used to inform the design of a new syphilis self-testing model, which will be implemented alongside the traditional facility-based testing service to increase syphilis screening among MSM in China. We anticipate that the model will increase early syphilis diagnosis and enhance syphilis continuum of care and treatment for MSM, and ultimately reduce the spread of syphilis and HIV transmission among MSM and other individuals within their sexual networks in China.
