# Supplementary material for: Expanding syphilis test uptake using rapid dual self-testing for syphilis and HIV among men who have sex with men in China: A multiarm randomized controlled trial
Source: PLoS Med. 2022 Mar 2;19(3):e1003930. doi: 10.1371/journal.pmed.1003930 (PMC8890628; doi:10.1371/journal.pmed.1003930)

# Methods and results for the economic evaluation

Table S6 summarizes the unit costs included in the decision tree model using a healthcare provider perspective and time horizon of 6 months (i.e. the study duration). Table S7 shows the unit cost per person tested and cost per person diagnosed, disaggregated by fixed costs and variable costs. To test the uncertainties of these unit costs in the model (Figure S3), we used gamma distributions for cost parameters and beta distributions for probability parameters. The model captures the main events within the trial (i.e. the decision of being in one of the three trial arms, and the chance or probability of testing for syphilis within the trial period, and the chance or probability of being tested positive for syphilis). Figures S4, S5, S7 and S8 summarizes the results from univariate sensitivity analyses showing that the factors that influenced the incremental cost the most. Figures S6 and S9 summarizes the probabilistic sensitivity analyses in an acceptability curve.

# Table A. Cost items included.

| **Variable costs** | **Unit cost (Yuan)** |
| --- | --- |
| Packaging | 2.8 |
| Instruction booklet | 1 |
| Syphilis test kit | 28 |
| Blood taking needle | 0.45 |
| Woundplast | 0.076 |
| Alcohol pad | 0.059 |
| Information card | 0.4 |
| Result upload card | 0.4 |
| Sexual health services list | 0.2 |
| Lottery-based incentive | 100 |
| Delivery cost of syphilis kits | 18.76 |
| Syphilis confirmation and treatment | 200 |
| HIV confirmation | 150 |
| **Fixed costs** | **Per month** |
| Staff time | 3000 per person |
| Internet | 200 |
| Utilities | 50 |
| Property management fee | 80 |
| Telephone bill | 50 |
| WenJuanXing platform rent for data collection | 240 |
| Building rental | 2000 |
| Computers | 4000 |
| Desks | 502 |
| Desk chairs | 274 |
|  |  |
| *Crowdsourced activities* | **Unit cost** |
| Platform construction | 5000 |
| Network promotional materials | 3000 |
| Prize | 6000 |
| Judging fee for experts | 1500 |
| Staff time | 1600 |

# Table B. Unit costs of standard-of-care, syphilis self-testing, and lottery-incentivized syphilis self-testing.

| Tests performed |  | Distribution |
| --- | --- | --- |
| - SOC | 10 Facility  12 SST | Beta |
| - SST | 4 Facility  122 SST | Beta |
| - Lottery | 3 Facility  140 SST | Beta |
| Syphilis diagnosed |  |  |
| - SOC | 1 | Beta |
| - SST | 7 | Beta |
| - Lottery | 8 | Beta |
| Fixed cost | Unit cost per person in each study arm (USD, 2020) |  |
| - SOC | 4.45 | Gamma |
| - SST | 4.31 | Gamma |
| - Lottery | 5.32 | Gamma |
| Variable cost |  |  |
| - SOC | 12.21 | Gamma |
| - SST | 15.52 | Gamma |
| - Lottery | 16.81 | Gamma |
| - Managing syphilis | 28.74 | Gamma |

SOC = standard-of-care; SST = syphilis self-testing; Lottery = lottery-incentivized syphilis self-testing.

# Figure A. Decision tree model.


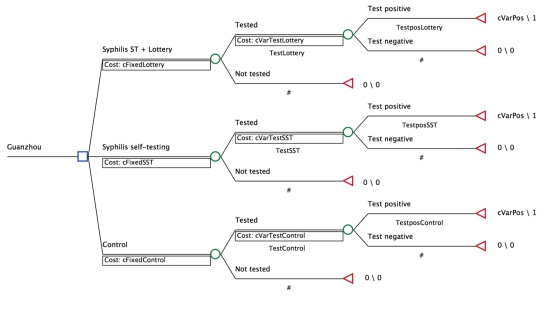


# Figure B. Tornado plot of the incremental cost per person tested for syphilis self-testing compared to standard-of-care.


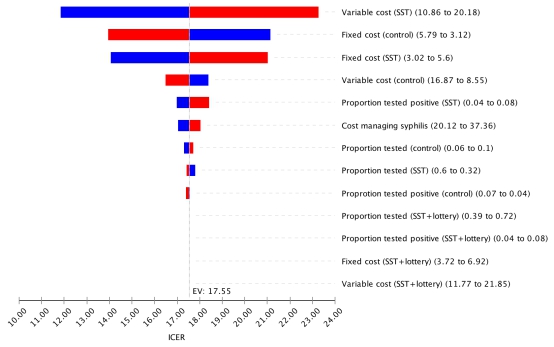


# Figure C. Cost-effectiveness acceptability curve for cost per person tested.

#
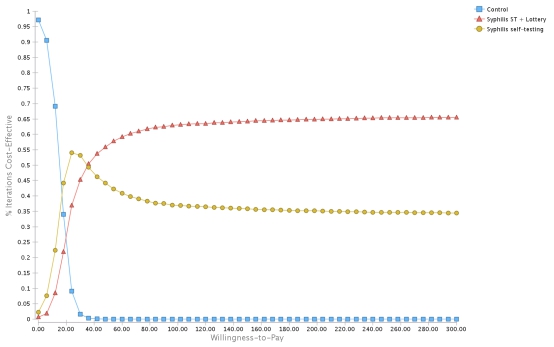


# Figure D. Tornado plot of incremental cost per person tested for lottery-incentivized syphilis self-testing compared to syphilis self-testing.


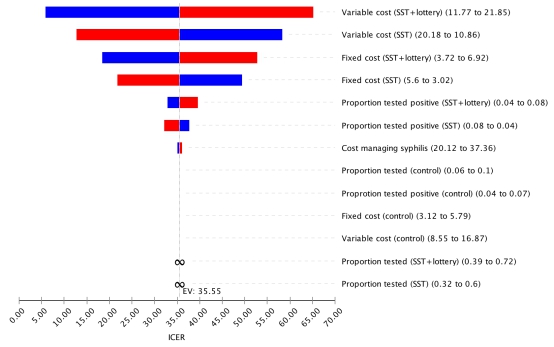


**Figure E. Tornado plot of incremental cost per person diagnosed for syphilis self-testing compared to standard-of-care.**


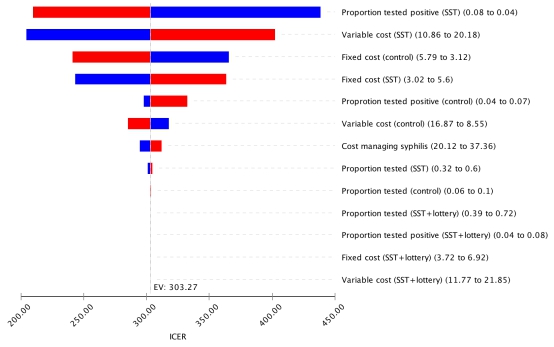


# Figure F. Tornado plot of incremental cost per person diagnosed for lottery-incentivized syphilis self-testing compared to syphilis self-testing.


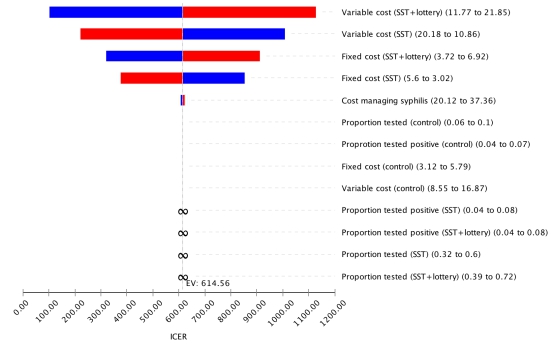


# Figure G. Cost-effectiveness acceptability curve for cost per person diagnosed.


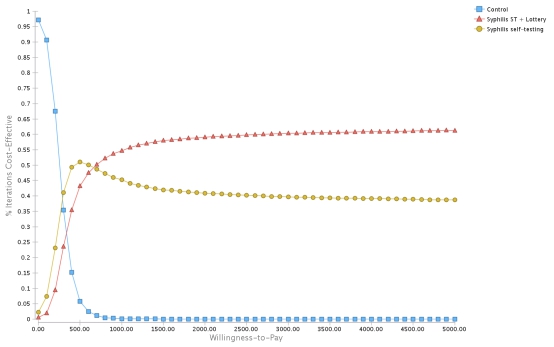

Supplement: S1 Appendix — Table A: Cost items included. Table B: Unit costs of SOC, SST, and lottery incentivized SST. Fig A: Decision tree model. Fig B: Tornado plot of the incremental cost per person tested for SST compared to SOC. Fig C: Cost-effectiveness acceptability curve for cost per person tested. Fig D: Tornado plot of incremental cost per person tested for lottery incentivized SST compared to SST. Fig E: Tornado plot of incremental cost per person diagnosed for SST compared to SOC. Fig F: Tornado plot of incremental cost per person diagnosed for lottery incentivized SST compared to SST. Fig G: Cost-effectiveness acceptability curve for cost per person diagnosed. SOC, standard of care; SST, syphilis self-testing. (DOC) [file pmed.1003930.s004.doc]
