# Supplementary material for: Expanding syphilis test uptake using rapid dual self-testing for syphilis and HIV among men who have sex with men in China: A multiarm randomized controlled trial
Source: PLoS Med. 2022 Mar 2;19(3):e1003930. doi: 10.1371/journal.pmed.1003930 (PMC8890628; doi:10.1371/journal.pmed.1003930)
Supplement: S1 Text — SST, syphilis self-testing. (DOCX) [file pmed.1003930.s005.docx]

# Web-link to an instructional video

https://www.alere.com/en/home/support/product-demos/sd-bioline-hiv-syphilis-duo.html
