# Supplementary figures and images for: Expanding syphilis test uptake using rapid dual self-testing for syphilis and HIV among men who have sex with men in China: A multiarm randomized controlled trial
Source: PLoS Med. 2022 Mar 2;19(3):e1003930. doi: 10.1371/journal.pmed.1003930 (PMC8890628; doi:10.1371/journal.pmed.1003930)

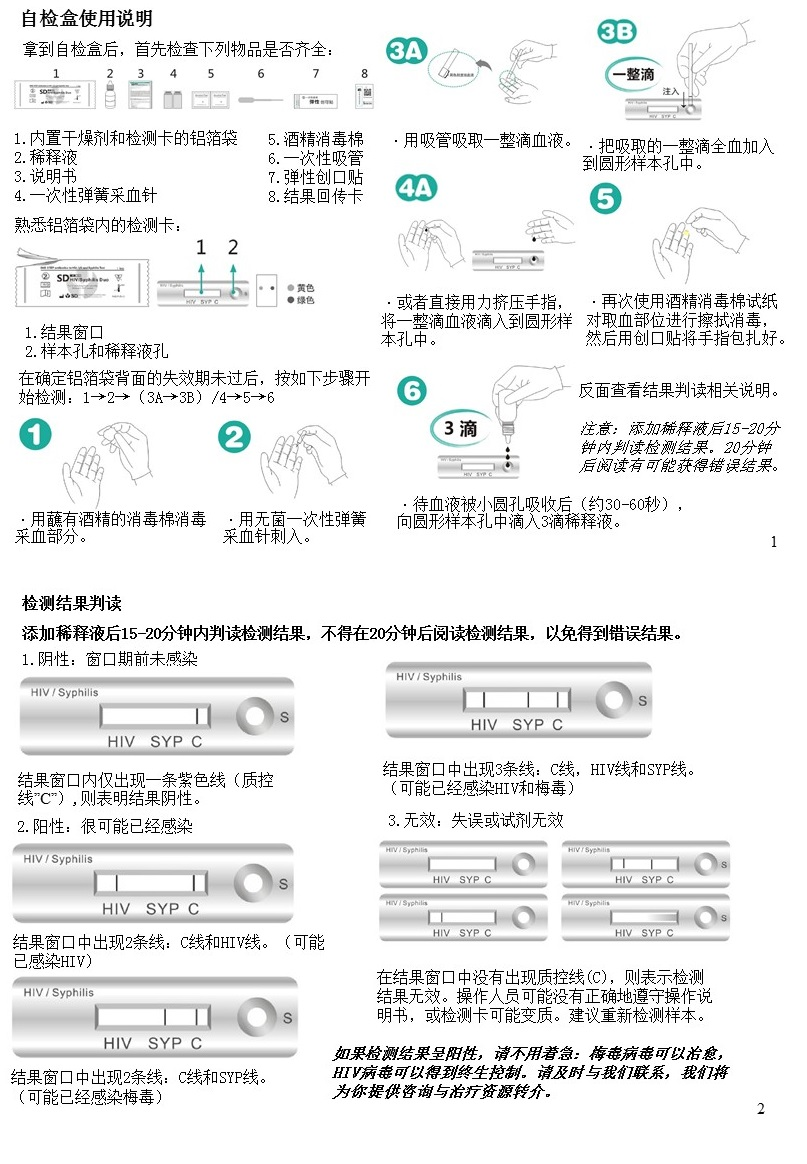

Supplement: S1 Fig — SST, syphilis self-testing. (TIF) [file pmed.1003930.s006.tif]

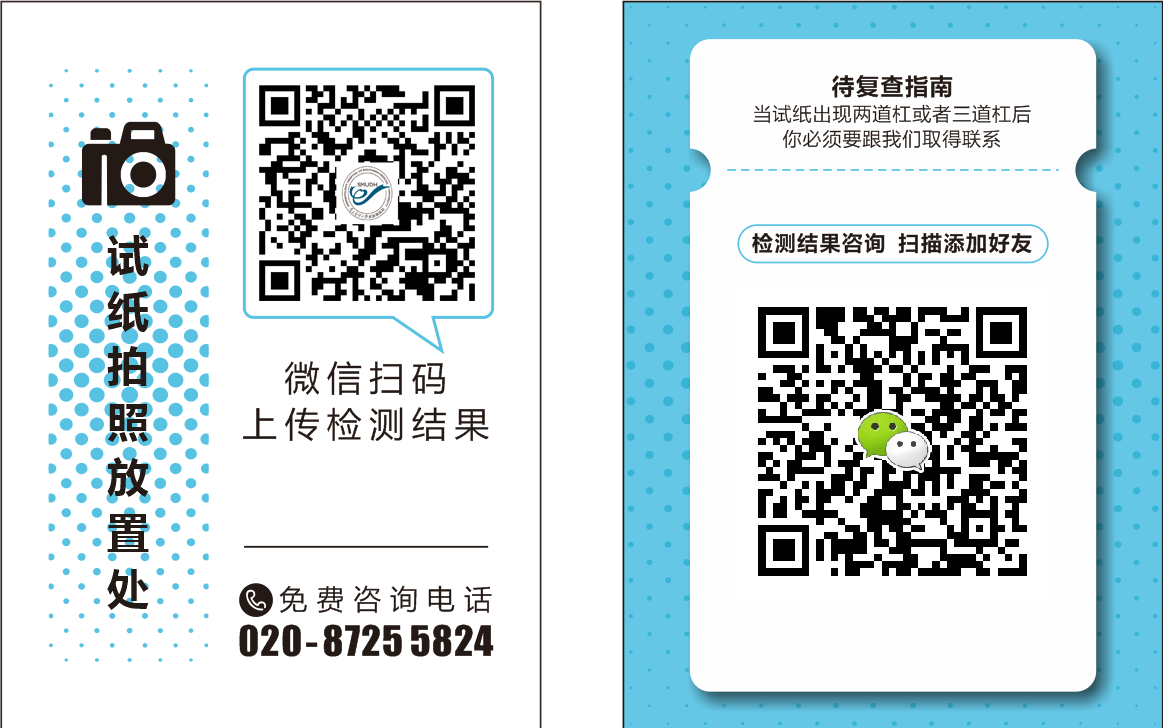

Supplement: S2 Fig — (TIF) [file pmed.1003930.s007.tif]

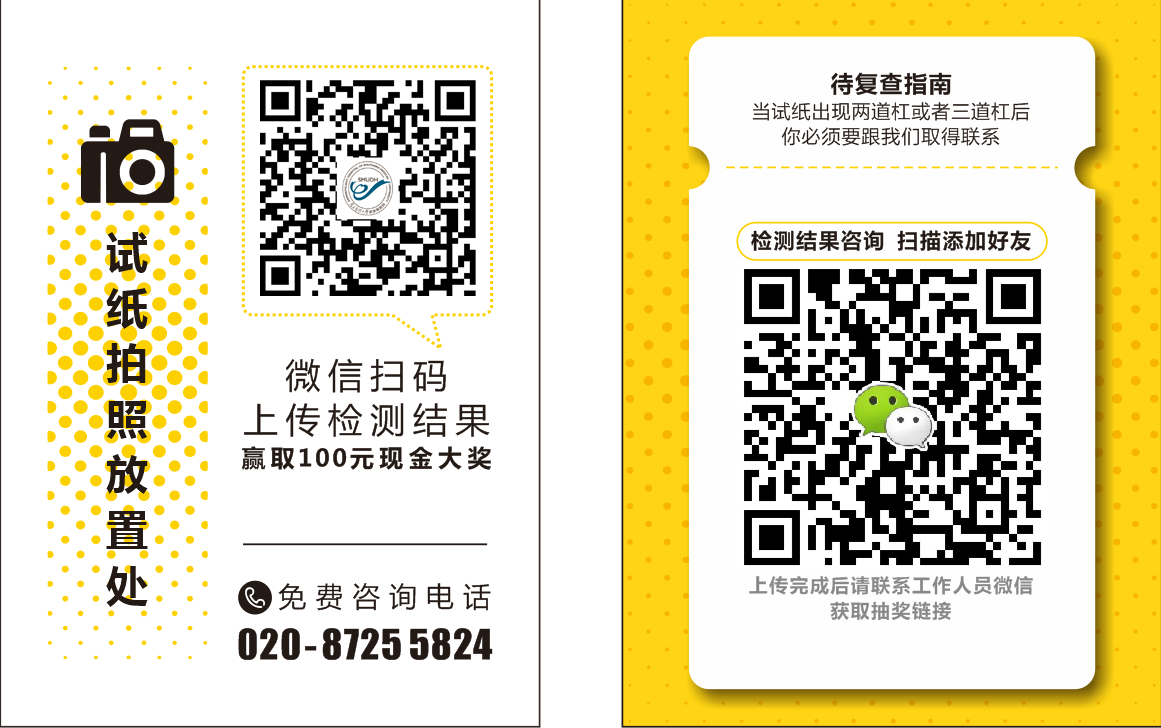

Supplement: S3 Fig — (TIF) [file pmed.1003930.s008.tif]
