# Supplementary material for: Expanding syphilis test uptake using rapid dual self-testing for syphilis and HIV among men who have sex with men in China: A multiarm randomized controlled trial
Source: PLoS Med. 2022 Mar 2;19(3):e1003930. doi: 10.1371/journal.pmed.1003930 (PMC8890628; doi:10.1371/journal.pmed.1003930)
Supplement: S1 Table — (DOCX) [file pmed.1003930.s009.docx]

# S1 Table. Participants’ geographic distribution.

| **Region** | **Provinces/Municipality/Autonomous region** | **City** | **Number** | **Lockdown or not during the trial** |
| --- | --- | --- | --- | --- |
| North | Beijing | Beijing | 6 | No |
|  | Tianjin | Tianjin | 1 | No |
|  | Hebei | Shijiazhuang | 1 | No |
|  | Inner Mongolia | Ordos | 1 | No |
| East | Shanghai | Shanghai | 1 | No |
|  | Jiangsu | Changzhou | 1 | No |
|  |  | Nanjing | 4 | No |
|  |  | Zhenjiang | 1 | No |
|  |  | Suzhou | 2 | No |
|  |  | Xuzhou | 1 | No |
|  | Zhejiang | Hangzhou | 23 | No |
|  |  | Jiaxing | 1 | No |
|  |  | Ningbo | 14 | No |
|  |  | Quzhou | 2 | No |
|  |  | Wenzhou | 10 | No |
|  |  | Jinhua | 3 | No |
|  |  | Taizhou | 5 | No |
|  |  | Lishui | 2 | No |
|  |  | Zhoushan | 1 | No |
|  |  | Shaoxing | 2 | No |
|  | Anhui | Anqing | 3 | No |
|  |  | Bengbu | 5 | No |
|  |  | Fuyang | 4 | No |
|  |  | Hefei | 13 | No |
|  |  | Huaibei | 5 | No |
|  |  | Wuhu | 4 | No |
|  |  | Huainan | 5 | No |
|  |  | Suzhou | 1 | No |
|  |  | Huangshan | 2 | No |
|  |  | Haozhou | 2 | No |
|  |  | Chuzhou | 3 | No |
|  |  | Tongling | 1 | No |
|  |  | Xuancheng | 4 | No |
|  | Jiangxi | Nanchang | 1 | No |
|  |  | Yichun | 1 | No |
|  |  | Jiujiang | 1 | No |
|  | Shandong | Dezhou | 1 | No |
|  |  | Heze | 1 | No |
|  | Fujian | Fuzhou | 11 | No |
|  |  | Quanzhou | 12 | No |
|  |  | Xiamen | 11 | No |
|  |  | Longyan | 3 | No |
|  |  | Zhangzhou | 1 | No |
|  |  | Sanming | 3 | No |
|  |  | Putian | 1 | No |
| Central | Henan | Zhengzhou | 18 | No |
|  |  | Luoyang | 3 | No |
|  |  | Puyang | 2 | No |
|  |  | Zhoukou | 2 | No |
|  |  | Xinyang | 5 | No |
|  |  | Anyang | 3 | No |
|  |  | Kaifeng | 4 | No |
|  |  | Nanyang | 4 | No |
|  |  | Xuchang | 1 | No |
|  |  | Xinxiang | 3 | No |
|  |  | Shangqiu | 1 | No |
|  |  | Zhumadian | 2 | No |
|  |  | Jiaozuo | 1 | No |
|  |  | Pingdingshan | 2 | No |
|  | Hubei | Wuhan | 2 | Yes |
|  |  | Xiantao | 2 | Yes |
|  |  | Xiangyang | 2 | No |
|  |  | Shiyan | 3 | No |
|  |  | Suizhou | 1 | No |
|  |  | Huangshi | 2 | No |
|  |  | Jingmen | 1 | No |
|  |  | Ezhou | 1 | Yes |
|  |  | Xianning | 2 | No |
|  |  | Yichang | 3 | No |
|  |  | Tianmen | 2 | No |
|  |  | Xiaogan | 2 | No |
|  |  | Jingzhou | 2 | No |
|  | Hunan | Changsha | 34 | No |
|  |  | Xiangtan | 1 | No |
|  |  | Shaoyang | 1 | No |
| South | Guangdong | Guangzhou | 9 | No |
|  |  | Shenzhen | 7 | No |
|  |  | Foshan | 2 | No |
|  |  | Jiangmen | 5 | No |
|  |  | Shanwei | 2 | No |
|  | Guangxi | Nanning | 2 | No |
|  | Hainan | Haikou | 2 | No |
| Southwest | Sichuan | Chengdu | 40 | No |
|  |  | Mianyang | 1 | No |
|  |  | Leshan | 2 | No |
|  |  | Neijiang | 4 | No |
|  |  | Luzhou | 2 | No |
|  |  | Nanchong | 4 | No |
|  |  | Panzhihua | 1 | No |
|  |  | Dazhou | 1 | No |
|  |  | Deyang | 3 | No |
|  |  | Yaan | 1 | No |
|  |  | Bazhong | 1 | No |
|  |  | Meishan | 1 | No |
|  |  | Ziyang | 1 | No |
|  | Guizhou | Guiyang | 8 | No |
|  |  | Qiandongnan | 1 | No |
|  |  | Tongren | 1 | No |
|  |  | Anshun | 1 | No |
|  |  | Zunyi | 2 | No |
|  |  | Liupanshui | 2 | No |
|  |  | Bijie | 1 | No |
|  | Yunnan | Kunming | 8 | No |
|  |  | Lijiang | 2 | No |
|  |  | Honghe | 3 | No |
|  |  | Baoshan | 1 | No |
|  |  | Qujing | 5 | No |
|  |  | Wenshan | 1 | No |
|  |  | Dali | 1 | No |
|  |  | Puer | 1 | No |
|  |  | Zhaotong | 1 | No |
|  | Chongqing | Chongqing | 1 | No |
| Northwest | Shanxi | Xian | 13 | No |
|  |  | Xianyang | 2 | No |
|  |  | Weinan | 1 | No |
|  |  | Ankang | 1 | No |
|  |  | Baoji | 2 | No |
|  |  | Yulin | 1 | No |
|  |  | Shangluo | 1 | No |
|  |  | Hanzhong | 1 | No |
|  | Gansu | Longnan | 1 | No |
| Northeast | Liaoning | Shenyang | 2 | No |
|  | Jilin | Changchun | 1 | No |
|  | Heilongjiang | Harbin | 1 | No |
