# Supplementary material for: Expanding syphilis test uptake using rapid dual self-testing for syphilis and HIV among men who have sex with men in China: A multiarm randomized controlled trial
Source: PLoS Med. 2022 Mar 2;19(3):e1003930. doi: 10.1371/journal.pmed.1003930 (PMC8890628; doi:10.1371/journal.pmed.1003930)
Supplement: S2 Table — RCT, randomized controlled trial; SST, syphilis self-testing. (DOCX) [file pmed.1003930.s010.docx]

# S2 Table. Baseline characteristics of study participants stratified by loss-to-follow-up in the Syphilis Self-Testing Randomized Controlled Trial in China in 2020.

|  | **Lost-to-follow-up (n=36)^a^** | **Completed at least one follow-up survey (n=415)^a^** | ***p*-value^b^** |
| --- | --- | --- | --- |
| **Age (years)** | | | 0.134 |
| ≤30 | 25/36 (69.4) | 333/415 (80.2) |  |
| >30 | 11/36 (30.6) | 82/415 (19.8) |  |
| Mean (SD) | 27.0 (7.6) | 25.5 (6.2) |  |
| **Marital status** | | | 0.203 |
| Never married | 29/36 (80.6) | 364/415 (87.7) |  |
| Ever married | 7/36 (19.4) | 51/415 (12.3) |  |
| **Annual income (US$)** | | | 0.024 |
| <3000 | 1/36 (2.8) | 84/415 (20.2) |  |
| 3000-6000 | 10/36 (27.8) | 75/415 (18.1) |  |
| 6001-9500 | 12/36 (33.3) | 130/415 (31.3) |  |
| 9501-12500 | 5/36 (13.9) | 73/415 (17.6) |  |
| ≥12501 | 8/36 (22.2) | 53/415 (12.8) |  |
| **Highest education** | | | 0.348 |
| High school or below | 8/36 (22.2) | 126/415 (30.4) |  |
| College or beyond | 28/36 (77.8) | 289/415 (69.6) |  |
| **Sexual orientation** | | | 0.574 |
| Gay/homosexual | 24/36 (66.7) | 294/415 (70.8) |  |
| Bisexual or other | 12/36 (33.3) | 121/415 (29.2) |  |
| **Disclosure as MSM to family, friends, or health-care professionals** | | | 0.015 |
| Never | 25/36 (69.4) | 199/415 (48.0) |  |
| Ever | 11/36 (30.6) | 216/415 (52.1) |  |
| **Anal sex without use of condom in the past 3 months** | | | 0.817 |
| No | 5/36 (13.9) | 70/415 (16.9) |  |
| Yes | 31/36 (86.1) | 345/415 (83.1) |  |
| **Ever tested for HIV** | | | 0.093 |
| No | 16/36 (44.4) | 126/415 (30.4) |  |
| Yes | 20/36 (55.6) | 289/415 (69.6) |  |
| **Ever tested for syphilis** | | | 0.828 |
| No | 28/36 (77.8) | 332/415 (80.0) |  |
| Yes | 8/36 (22.2) | 83/415 (20.0) |  |
| **Ever used a syphilis self-test** | | | 0.757 |
| No | 34/36 (94.4) | 381/415 (91.8) |  |
| Yes | 2/36 (5.6) | 34/415 (8.2) |  |

# ^a^Data are n/N (%) unless otherwise indicated. ^b^*p*-value computed using a two-sided Fisher’s exact test.
