# Supplementary material for: Expanding syphilis test uptake using rapid dual self-testing for syphilis and HIV among men who have sex with men in China: A multiarm randomized controlled trial
Source: PLoS Med. 2022 Mar 2;19(3):e1003930. doi: 10.1371/journal.pmed.1003930 (PMC8890628; doi:10.1371/journal.pmed.1003930)
Supplement: S3 Table — (DOCX) [file pmed.1003930.s011.docx]

# S3 Table. Sensitivity analysis on the uptake of syphilis testing among all participants.

|  | **Original** | **Worst Case^a^** | **Best Case^b^** | | **MI^c^** | **Risk Difference (95 % Wald CI)^d^** | | |
| --- | --- | --- | --- | --- | --- | --- | --- | --- |
|  | **n/N (%)** | **n/N (%)** | **n/N (%)** | **n (rounded)/N (%)** | | **Original vs Worst** | **Original vs Best** | **Original vs MI** |
| **Standard-of-care** | | | | | | | | |
| 3-month | 7/131 (5.3) | 7/150 (4.7) | 26/150 (17.3) | | 13/150 (8.7) | 0.7 (-4.4, 5.8) | -12.0 (-19.2, -4.8) | -3.5 (-10.2, 3.3) |
| 6-month | 14/129 (10.9) | 14/150 (9.3) | 35/150 (23.3) | | 20/150 (13.3) | 1.5 (-5.6, 8.6) | -12.5 (-21.1, -3.8) | -2.2 (-10.7, 6.3) |
| overall | 20/136 (14.7) | 20/150 (13.3) | 34/150 (22.7) | | 28/150 (18.7) | 1.4 (-6.7, 9.4) | -8.0 (-16.9, 1.0) | -3.9 (-13.4, 5.7) |
| **Standard SST** | | | | | | | | |
| 3-month | 74/137 (54.0) | 74/151 (49.0) | 88/151 (58.3) | | 79/151 (52.3) | 5.0 (-6.5, 16.6) | -4.3 (-15.7, 7.2) | 1.9 (-9.9, 13.7) |
| 6-month | 51/139 (36.7) | 51/151 (33.8) | 63/151 (41.7) | | 54/151 (33.8) | 2.9 (-8.1, 13.9) | -5.0 (-16.3, 6.2) | 0.7 (-10.7, 12.0) |
| overall | 90/142 (63.4) | 90/151 (59.6) | 99/151 (65.6) | | 95/151 (62.9) | 3.8 (-7.4, 14.9) | -2.2 (-13.2, 8.8) | 0.7 (-10.7, 12.2) |
| **Lottery incentivized SST** | | | | | | | | |
| 3-month | 72/134 (53.7) | 72/150 (48.0) | 88/150 (58.7) | | 78/150 (52.0) | 5.7 (-5.9, 17.4) | -4.9 (-16.5, 6.6) | 2.0 (-10.0, 13.9) |
| 6-month | 69/126 (54.8) | 69/150 (46.0) | 93/150 (62.0) | | 77/150 (51.3) | 8.8 (-3.0, 20.6) | -7.2 (-18.9, 4.4) | 3.5 (-8.7, 15.7) |
| overall | 90/137 (65.7) | 90/150 (60.0) | 103/150 (68.7) | | 96/150 (64.0) | 5.7 (-5.5, 16.9) | -3.0 (-13.9, 7.9) | 1.5 (-10.1, 13.0) |

Sensitivity analyses involved the replacement of missing values with the worst value ^a^ (not tested for syphilis) and best value ^b^ (tested for syphilis) in the observed data. ^c^For multiple imputation (MI), Rubin’s rule was used to compute a pooled estimate for the risk differences and standard errors. ^d^Risk differences expressed as a percentage.
