# Supplementary material for: Expanding syphilis test uptake using rapid dual self-testing for syphilis and HIV among men who have sex with men in China: A multiarm randomized controlled trial
Source: PLoS Med. 2022 Mar 2;19(3):e1003930. doi: 10.1371/journal.pmed.1003930 (PMC8890628; doi:10.1371/journal.pmed.1003930)
Supplement: S4 Table — (DOCX) [file pmed.1003930.s012.docx]

S4 Table. Newly identified syphilis infections and linkage to care among men who had syphilis testing during the trial.

|  | **Standard-of-care** | **Standard SST group** | **Lottery incentivized SST group** |
| --- | --- | --- | --- |
|  | **n/N (%)** | **n/N (%)** | **n/N (%)** |
| **Testers with reactive result^a^** | | | |
| Month 0-3 | 0/7 (0.0) | 1/74 (1.4) | 1/72 (1.4) |
| Month 4-6 | 1/14 (7.1) | 1/51 (2.0) | 1/69 (1.4) |
| overall | 1/20 (5.0) | 2/90 (2.2) | 2/90 (2.2) |
| **Testers with reactive result received confirmatory testing^b^** | | | |
| Month 0-3 | NA | 1/1 (100.0) | 1/1 (100.0) |
| Month 4-6 | 1/1 (100.0) | 1/1 (100.0) | 1/1 (100.0) |
| overall | 1/1 (100.0) | 2/2 (100.0) | 2/2 (100.0) |
| **Testers with reactive result received treatment^b^** | | | |
| Month 0-3 | NA | 1/1 (100.0) | 1/1 (100.0) |
| Month 4-6 | 1/1 (100.0) | 1/1 (100.0) | 1/1 (100.0) |
| overall | 1/1 (100.0) | 2/2 (100.0) | 2/2 (100.0) |

^a^These results were verified using photo-verification. ^b^These results were self-reported.
