# Supplementary material for: Expanding syphilis test uptake using rapid dual self-testing for syphilis and HIV among men who have sex with men in China: A multiarm randomized controlled trial
Source: PLoS Med. 2022 Mar 2;19(3):e1003930. doi: 10.1371/journal.pmed.1003930 (PMC8890628; doi:10.1371/journal.pmed.1003930)
Supplement: S5 Table — (DOCX) [file pmed.1003930.s013.docx]

# S5 Table. Syphilis testing by mode: self-test, facility-based test, or both.

|  | **Standard-of-care** | | **Standard SST** | **Lottery incentivized SST** | | ***p*-value^a^** | |
| --- | --- | --- | --- | --- | --- | --- | --- |
|  | **n/N (%)** | | | | | ***SOC vs SST*** | ***SOC vs Lottery*** |
| **3-month** | | | | | | 0.0003 | 0.0001 |
| Self-test | 3/7 (42.9) | 72/74 (97.3) | | | 70/72 (97.2) |  |  |
| Facility-based test | 4/7 (57.1) | 2/74 (2.7) | | | 1/72 (1.4) |  |  |
| Both | 0/7 (0.0) | 0/74 (0.0) | | | 1/72 (1.4) |  |  |
| **6-month** | | | | | | 0.0004 | <0.0001 |
| Self-test | 8/14 (57.1) | 49/51 (96.1) | | | 68/69 (98.6) |  |  |
| Facility-based test | 5/14 (35.7) | 1/51 (2.0) | | | 0/69 (0.0) |  |  |
| Both | 1/14 (7.1) | 1/51 (2.0) | | | 1/69 (1.5) |  |  |
| **Overall** | | | | | | <0.0001 | <0.0001 |
| Self-test | 11/20 (55.0) | 86/90 (95.6) | | | 87/90 (96.7) |  |  |
| Facility-based test | 8/20 (40.0) | 1/90 (1.1) | | | 0/90 (0.0) |  |  |
| Both | 1/20 (5.0) | 3/90 (3.3) | | | 3/90 (3.3) |  |  |

*^a^p*-value computed using a two-sided Fisher’s exact test. SOC = standard-of-care; SST = syphilis self-testing; Lottery = lottery-incentivized syphilis self-testing.
