# Supplementary material for: Expanding syphilis test uptake using rapid dual self-testing for syphilis and HIV among men who have sex with men in China: A multiarm randomized controlled trial
Source: PLoS Med. 2022 Mar 2;19(3):e1003930. doi: 10.1371/journal.pmed.1003930 (PMC8890628; doi:10.1371/journal.pmed.1003930)
Supplement: Attachment — Submitted filename: Response to Editor and Reviewer-25Oct2021.docx [file pmed.1003930.s014.docx]

Dear PLOS Medicine Editors

Thank you for the opportunity to respond to the helpful reviewer comments. We have used the comments to strengthen the paper. Find attached a tracked changes and a clean version of the manuscript. Below are point-by-point responses and italicized text refers to the new text within the manuscript.

Thank you for your consideration.

Sincerely,

Wang Cheng, Anne Marie Weideman, Jason Ong, and Joe Tucker on behalf of the co-authors

**Response to the editors**

***Comment 1:***

1)  Abstract:
a) Please structure your abstract using the PLOS Medicine headings (Background, Methods and Findings, Conclusions).
i) Please combine Background and Objective into one section, “Background.”
ii) Please combine the Design, Setting, Participants, Measurements, Results and Limitations sections into one section, “Methods and findings”. Please ensure that all numbers presented in the abstract are present and identical to numbers presented in the main manuscript text.
b) Please report your abstract according to CONSORT for abstracts: [http://www.consort-statement.org/extensions?ContentWidgetId=562](http://www.consort-statement.org/extensions?ContentWidgetId=562#inbox/_blank)
c) Please indicates the dates during which study enrollment and follow up occurred.
d) Please provide the number of participants in each group.
e) Please provide the number of participants lost to follow up in each group.
f) Please state that the analysis was intention to treat.
g) Please quantify the main results (with p values in addition to 95% CI).
h) Please include a summary of adverse events if these were assessed in the study

***Response:*** Thanks for the guidance. We have revised the abstract as per request below.

***(Page 2) Abstract***

***Background***

*Low syphilis testing uptake is a major public health issue among men who have sex with men (MSM) in many low- and middle- income countries. Syphilis self-testing (SST) may complement and extend facility-based testing. We aimed to evaluate the effectiveness and costs of providing SST on increasing syphilis testing uptake among MSM in China.*

***Methods and findings***

*An open label three-arm randomized controlled trial was conducted between 7 January 2020 and 17 July 2020. Men who were at least 18 years of age, had condomless anal sex with men in the past year, reported not testing for syphilis in the last six months, and had a stable residence with mailing addresses were recruited from 124 cities in 26 Chinese provinces. Using block randomization with blocks of size 12, enrolled participants were randomly assigned (1:1:1) into three arms: standard of care arm, standard syphilis self-testing arm, and lottery incentivized syphilis self-testing arm (1 in 10 chance to win $15 if they tested for syphilis). The primary outcome was the proportion of participants who tested for syphilis during the trial period and confirmed with photo-verification, and between arm comparisons were estimated with risk differences. Analyses were performed on a modified intention to treat basis: participants were included in the complete case analysis if they completed at least one follow-up survey. The SD BIOLINE Syphilis/HIV Duo rapid test kit was used. A total of 451 men were enrolled. 136 (90·7%, 136/150) in the standard of care arm, 142 (94·0%, 142/151) in the standard of SST arm, and 137 (91·3%, 137/150) in the lottery incentivized SST arm were included in the final analysis. The proportion of men who had at least one syphilis test during the trial period was 63.4% (p=0.001, 95% CI: 55.5 to 71.3%) in the standard SST arm, 65.7% (p=0.0002, 95% CI: 57.7 to 73.6%) in the lottery-incentivized SST arm, and 14.7% (p<0.0001, 95% CI: 8.8 to 20.7%) in the standard-of-care arm. The estimated risk difference between the standard SST and standard-of-care arm was 48.7% (p<0.0001, 95% CI: 37.8 to 58.4%). The majority (78.5%, p<0.0001, 95% CI: 72.7 to 84.4%) of syphilis self-testers reported never testing for syphilis. The cost per person tested was $26.55 for standard SST, $28.09 for the lottery incentivized SST, and $66.19 for the standard-of-care. No study-related adverse events were reported during the study duration. Limitation was that the impact of COVID restrictions may have accentuated demand for decentralized testing. The trial is registered with the Chinese Clinical Trial Registry, number ChiCTR1900022409. The study design and results were reported according to CONSORT 2010 guidelines.*

***Conclusions***

*Compared to standard-of-care, providing SST significantly increased the proportion of MSM testing for syphilis in China, and was cheaper (per person tested).*

***Comment 2:***  Data availability statement: If the data are owned by a third party but freely available upon request, please note this and state the owner of the data set and contact information for data requests (web or email address). Note that a study author cannot be the contact person for the data.

***Response:*** We have revised the Data availability statement as below: De-identified data cannot be made open accessed because of the IRB decision to ensure patient confidentiality. Permission can be requested by contacting the Dermatology Hospital of Southern Medical University (gdpfzx@vip.163.com).

***Comment 3:***   Author summary - At this stage, we ask that you reformat your non-technical Author Summary. The Author Summary should immediately follow the Abstract in your revised manuscript. This text is subject to editorial change and should be distinct from the scientific abstract. The summary should be accessible to a wide audience that includes both scientists and non-scientists.

***Response:*** We have added the Author Summary following with the Abstract in the revised manuscript.

# *(Page 4) Author Summary*

## *Why Was This Study Done?*

- *MSM have a high burden of syphilis while testing coverage remains low.*
- *SST could be useful to help expend syphilis testing among MSM.*
- *This study is the first RCT to evaluate the effectiveness and cost of SST.*

## *What Did the Researchers Do and Find?*

- *We recruited 451 MSM participants in a three-arm randomized controlled trial from 124 cities in Chinese 26 provinces between 7 January 2020 and 17 July 2020.*
- *Overall, the proportion of men who had at least one syphilis test during the trial period was 63.4% (p=0.001, 95% CI: 55.5 to 71.3%) in the standard SST arm, 65.7% (p=0.0002, 95% CI: 57.7 to 73.6%)* *in the lottery-incentivized SST arm, and 14.7% (p<0.0001, 95% CI: 8.8 to 20.7%) in the standard of care arm. The majority (78.5%, p<0.0001, 95% CI: 72.7 to 84.4%) of syphilis self-testers reported that the self-test was their first syphilis test.*
- *The cost per person tested was $66.19 for the standard-of-care arm, $26.55 for standard SST and $28.09 for the lottery incentivized SST arm.*

## *What Do These Findings Mean?*

- *Our randomized controlled trial and economic evaluation strengthens the evidence for syphilis self-testing programs among MSM.*
- *The interpretation of our study's findings might be affected by the COVID restrictions, which may have accentuated demand for decentralized testing.*
- *Future studies are needed to enhance linkage to clinical and public health services after an individual uses a self-test kit.*

***Comment 4:***  Thank you for completing the CONSORT checklist. Please align the reporting of your manuscript with the checklist and replace the page numbers with paragraph numbers per section (e.g. "Methods, paragraph 1"), since the page numbers of the final published paper may be different from the page numbers in the current manuscript.

***Response:*** We have aligned the reporting of our manuscript with the checklist and replaced the page numbers with paragraph numbers per section (see the Appendix).

***Comment 5:***   Please also report your study in line with the CONSERVE 2021 Statement checklist, a guideline for reporting trials which have been disrupted by COVID-19 and other circumstances. The guideline can be found at [https://pubmed.ncbi.nlm.nih.gov/34152382/](https://pubmed.ncbi.nlm.nih.gov/34152382/#inbox/_blank) . In the methods, please indicate the study has been reported according to the CONSERVE 2021 statement, and provide the checklist as a supplementary file.

***Response:*** We have reported our study in line with the CONSERVE 2021 Statement checklist, indicated the study has been reported according to the CONSERVE 2021 statement, and provide the checklist as a supplementary file in the third paragraph of the Methods section in the revised manuscript.

***Comment 6:***  Please specify whether informed consent was obtained and whether it was written or oral.

***Response:*** We have specified the process of obtaining the informed consent in the revised manuscript (see the sixth paragraph of Methods section).

*(Page 8) All participants provided online informed consent before the baseline survey by clicking on a “button” indicating that the participant has read the consent form and agrees to participate.*

***Comment 7:***  Please remove the “Role of the funding source”, “Declaration of interests”, and “Data sharing” from the main text. This information is captured in the metadata obtained in the submission form.

***Response:*** We have removed the “Role of the funding source”, “Declaration of interests”, and “Data sharing” from the main text in the revised manuscript.

***Comment 6:***  In the Methods and Results section:
a)      Please provide 95% CIs and p values for estimates in the main text and tables
b)      When a p value is given, please specify the statistical test used to determine it.

***Response:*** We have added 95% CIs and p-values for estimates in the main text and tables, and specified the statistical test used to determine the p-value in the Methods of the revised manuscript.

***Comment 7:***  Figures and tables:
a) Please rename Figure 2, CONSORT flow chart
b) Please indicate in the figure caption the meaning of the whiskers in Figures
c) Please provide definitions for the following abbreviations: IC, AEs, EU/mL
d) Please ensure that Figure 1 complies with our figure requirement: [http://journals.plos.org/plosmedicine/s/figures](http://journals.plos.org/plosmedicine/s/figures#inbox/_blank).
e) Please confirm that the appropriate usage rights apply to the use of this Figure 1. Please see our guidelines for map images: [https://journals.plos.org/plosmedicine/s/figures#loc-maps](https://journals.plos.org/plosmedicine/s/figures#inbox/_blank)

***Response:*** Thanks for the guidance. a) We have renamed Figure 2 as “CONSORT flow chart”. b) We do not have whiskers in our Figures. c) We did not use these abbreivations. d) We have checked the requirements and ensure that the revised Figure 1 complies with the requirements. e) We confirm that the appropriate usage rights apply to the use of this Figure 1.

***Comment 8:***  References:
a) Please select the PLOS Medicine reference style in your citation manager. In-text reference call outs should be presented as follows noting the absence of spaces within the square brackets: "... countries [1,2]."
b) In the reference section, please ensure six names appear before et al.
c) Please ensure that journal name abbreviations consistently match those found in the National Center for Biotechnology Information (NCBI) databases. [https://journals.plos.org/plosmedicine/s/submission-guidelines#loc-references](https://journals.plos.org/plosmedicine/s/submission-guidelines#inbox/_blank).
d)  Please include access dates for all weblinks (e.g., Ref #32, and ensure that all weblinks are current and accessible.

***Response:*** Thanks for the guidance. We have revised the references according to the requirements in the revised manuscript.

***Comment 9:*** To help us extend the reach of your research, please provide any Twitter handle(s) that would be appropriate to tag, including your own, your coauthors’, your institution, funder, or lab.

***Response:*** Our co-author Twitter handles include the following: @JosephTucker, @DrJasonJOng, @sesh_global, @dr_michaelmarks

**Response to the Academic editor:**

***Comment 1:*** Please pay special attention to reviewer concerns regarding: 1) the “dual intervention and 2) drop-out rates and availability of generic information on those who did not proceed with the rest of the study.
The authors could address the first point (about dual HIV/syphilis testing) as a limitation to the study and just comment on its possible implications. The second point (very high rate of self-exclusion) is more problematic, as it does call into question the generalizability of the results. This needs to be addressed in the analysis.

***Response:*** Thanks for the editor’s guidance. 1. We have put the first point as a limitation to the study and comment on its possible implications in the third paragraph of the Discussion section. 2. In terms of this issue, we have provided potential reasons: 1) we recruited participants from a large MSM-oriented mobile social app - Blued (Danlan, Beijing, China). We designed a vivid invitation poster which was posted on Blued’s portal website and startup screen. The poster would jump out and last for four seconds when people entered the website to inform them that they would have a chance to get free syphilis self-testing kits if they participated in the study. There were many people who might be interested in getting free SST kits, but withdrew after clicking the poster (link) when they discovered that there was a survey requirement. Additionally, there might be people who just clicked the poster unintentionally. By February 2018, Blued had 40 million registered users globally, with 70% in Mainland China. Therefore, the number in those two cases above would not be small. 2) Repeat clicking of the link was also problematic. Our survey was restricted to one phone number and a single device (IP address). After the survey was completed, the phone number and IP address connected to that survey could not be used to access the survey again. However, if participants were unavailable to complete the survey in one sitting, they would need to renter the survey link several times in order to finish the entire survey. Additionally, some participants who finished the survey may attempt to complete the survey again to obtain a subsidy, although the system would tell them they could only participate one time. We have also discussed the generalizability issue in the third paragraph of the Discussion section in the manuscript.

***Comment 2:***  There is no discussion at all of the confidentiality/unintentional disclosure risk of requesting a syphilis test. I assume that there must be some stigma associated with STIs. It would improve the paper to learn something about this.

***Response:*** Thanks for the suggestion. Although we have already had such kind of information in the manuscript (see the sixth paragraph of the Methods section), we agree that there might be a risk of inadvertent disclosure of a requesting a syphilis test. We have added this to the limitation section (see the seventh paragraph of the Methods section).

*(Page 8)Eligible participants completed a baseline survey and were required to provide contact information including a cell phone number or a Wechat account to receive text messages, a shipping address for participants who applied for kits, and a preferred name, which were all stored in a password-protected file that a designated research assistant could only access.*

*(Page 18) Sixth, there might be a risk of inadvertent disclosure of requesting a syphilis test.*

***Comment 3:***  Related to point 1, there is no mention of the yield (% positive) for any of the arms. It's reasonable to guess that those actively seeking a test are at higher risk than those not. This could differ by arm; in any case it almost surely differs among those volunteering to participate and those not. The yield also affects the cost-effectiveness. This needs to be discussed, if not incorporated into the analysis.

**Response:** We have provided such information in the seventh paragraph of the Results section, and put the related figures into the Appendix. In terms of the cost-effectiveness, we agree that the yield has an impact on the cost-effectiveness analysis. This was assessed in our deterministic univariate sensitivity analyses. Specifically, in Supplementary Figure S4 and S5, we evaluated the impact of changes in the potential yield +/- 30%, demonstrating it was the fifth most sensitive variable that impacted the ICER.

*Of the 200 participants tested for syphilis, 191 (95.5%) men were syphilis self-testing and 150 (78.5%) men reported that the self-test was their first ever syphilis test. Fourteen (7.0%, 14/200) men were found to be infected with syphilis, five of whom (35.7%, 5/14) were newly identified through this study (one was in the standard-of-care arm, two in the standard SST arm, and two in the lottery incentivized arm), and all five reported receiving further confirmatory testing and treatment (Appendix p 10, Table S5). Additionally, among those who self-tested for syphilis, 18 (9.4%, 18/191) men were found to be infected with HIV, and eight men (44.4%, 8/18) were newly identified through this study. The proportion of men who conducted syphilis testing by different testing mode (self-testing versus facility-based testing) are summarized in the Appendix (Appendix p 8, Table S3).*

***Comment 4:***  The cost analysis did not capture the "full economic cost" as stated. It captured the financial cost to the provider. This is fine in terms of methods but a full economic evaluation should not be claimed. Including the cost of developing the lottery is odd, as this appears to be a research-specific cost that would not be incurred if the intervention were to be adopted for routine use. I would take that out.

***Response:*** Our costing plan was designed to be a full economic cost, i.e. including volunteer time and donated goods. However, the reviewer is technically correct that our final analysis did not have any costs related to volunteer time or donated goods, so is technically a financial cost to the provider. We have amended this in the seventh paragraph in the revised manuscript.

We have added further text to clarify the reason why we included the costs related to the crowdsourcing activity – this was a key activity related to the development of the intervention for this arm (that distinguished it from the SST arm). Specifically, the crowdsourcing activity generated the images used to promote the lottery. Future use of this intervention will also need to generate contextually appropriate images to encourage SST use with a lottery incentive. Thus, this separate cost is important to account for in the lottery-incentivized SST arm.

Changes:

*(Page 9)An open crowdsourcing contest determined the procedure of the lottery draw and the images that would be shown to men in the lottery incentivized SST arm. The crowdsourcing contest resulted in 14 lottery schemes from which the winning entries were then used to develop the lottery draw program in this trial.*

*(Page 12)Economic evaluation*

*We used a microcosting approach to estimate the* ***financial*** *cost from a health provider perspective. Costs were collected alongside the trial and categorized as either fixed or variable.*

**Response to reviewer #1**

***Comment 1:*** Was a statistical analysis plan prepared for the trial before data were unblinded to the investigators? What led to the changes in the statistical analysis specified in the protocol (a GEE model)?

***Response:*** First, we thank the statistical reviewer for their insightful feedback that greatly improved the statistical presentation of our results.

To answer this first question, the statistical analysis plan was prepared before study personnel were unblinded and is presented in the published protocol. There was no separate analysis plan.

The generalized estimating equations (GEE) specified in the analysis plan were not needed since the testing outcome was coded as binary yes/no once per period. For example, in months 0-3, a patient was recorded as“yes”for testing if they tested at least once within this period and“no”for testing if they did not test. Thus, using a GEE would return the same result as a GLM since there is only one record per patient within the respective period.

***Comment 2:*** In the protocol, it was specified that if outcome measures were missing for 11-<20% of the participants, a sensitivity analysis with multiple imputation would be used. It looks based on Figure 2 that this is the case for the 6 months follow-up outcome. What was the results of this? For 3 months follow-up it is very close to 11% missing data - it would strengthen the reporting of the study if an appropriate MI procedure was completed with 3-month follow-up data as well.

***Response:*** We agree and have revised the Methods (see the second paragraph of the Statistical analysis) and Table S4 in the revised manuscript as follows:

*“**We also conducted sensitivity analyses on uptake of syphilis testing using non-responder imputation (NRI) to impute the worst- and best-case scenarios and multiple imputation (MI) by fully conditional specification (FCS) with logistic regression. For non-responder imputation, the sensitivity analyses involved replacing missing values with the worst value (not tested for syphilis) and best value (tested for syphilis) in the observed data. We assumed for the best value that all the men with missing follow-up data who applied for the syphilis self-testing kits received syphilis testing during the trial.* *For multiple imputation, Rubin’s rule was used to compute pooled estimates for the risk differences and standard errors.* *Compared to those who completed at least one follow-up survey, participants lost-to-follow-up differed on three demographic variables included in the logistic regression (Appendix p 6-7, Table S2): Annual income, disclosure as MSM to family, friends, or healthcare professionals, and ever tested for HIV.”*

***Comment 3:*** One thing to clarify - how would patients in standard care arm be able to provide a photo verification of testing? Are patients routinely given results via email/message or are they given at in-person appointments? Could this change the ascertainment of outcomes in this usual care group?

***Response:*** Patients in China who receive a syphilis test at a facility would receive their results by print notification at in-person appointments. Men photographed this notification and sent it to the study organizers. Only men who submitted photo-verification were provided with $1, so there was also a financial incentive to provide verification. This method of ascertainment would not change the usual flow of information following syphilis testing at a facility.

***Comment 4:*** Was there any generic information captured before consent about who did not proceed with the rest of the study? The drop-out rate at this point is fairly high and I was interested in the reasons why so many people dropped out at this point.

***Response:*** We were not able to capture the generic information of participants who withdrew the study before reading the consent. The potentials reasons might include: 1) Repeat clicking of the link. Our survey was restricted to one phone number and a single device (IP address). After the survey was completed, the phone number and IP address connected to that survey could not be used to access the survey again. However, if participants were unavailable to complete the survey in one sitting, they would need to reenter the survey link several times in order to finish the entire survey. Additionally, some participants who finished the survey may attempt to complete the survey again to obtain a subsidy, although the system would tell them they could only participate one time. 2) we recruited participants from a large MSM-oriented mobile social app - Blued (Danlan, Beijing, China). We designed a vivid invitation poster which was posted on Blued’s portal website and startup screen. The poster would jump out and last for four seconds when people entered into the website to inform them that they would have a chance to get free syphilis self-testing kits if they participated in the study. There were many people who might be interested in getting free SST kits, but withdrew after clicking the poster (link) when they discovered that there was a survey requirement. Additionally, there might be people who just clicked the poster unintentionally. By February 2018, Blued had 40 million registered users globally, with 70% in Mainland China. Therefore, the number in those two cases above would not be small.

***Comment 5:*** P2, Abstract, Results. It's unclear at first glance what the '48.7%' refers to - maybe rephrasing as 'absolute difference in proportions' would be clearer?  In tables, it's referred to as risk difference which would also work.

***Response:*** We agree with this helpful comment, and we have modified from ‘difference in proportions’ to ‘risk difference’ to describe ‘48.7%’ (see the second paragraph of the Abstract).

***Comment 6:*** P5, Recruitment. Just to clarify - this was advertised on Blued, but users could share the link via other social media?

***Response:*** Yes, users could share the link via other social media.

***Comment 7:***  P9, Sample Size. Setting the number of participants based on >99% looks a bit odd - was the sample size for the study fixed and then the power calculated after this had been decided? I don't have any objections to the sample size here, 120 per arm is not an outrageous number and the study arms are unlikely to have any detrimental effect on participants, it's just unusual to plan a sample size with power >0.9. The sample size information presented here is different to that in the protocol - what was the reason for the update?

***Response:***

We acknowledge deficiencies in the published protocol that required revised sample size calculations. To revise, we used a two-sided Pearson Chi-square test for difference in proportions to estimate the sample size. However, with this correction we realize that we enrolled more participants than was necessary to achieve at least 80% power. With this correction to our code, we were now powered at >99% to detect a difference in proportions between the standard-of-care and standard SST. However, since it is not typical to complete ‘post-hoc power calculations,’ we have removed this section from the manuscript and will refer readers to the published protocol.

***Comment 8:*** P9, General Analysis. 'modified intention to treat' is a variable term that I think is almost meaningless. It would be better to be straightforward and say exactly what the assumptions were, e.g. analysed as randomised to the extent we had at least one available visit, with list-wise deletion

***Response:*** Thank you for this suggestion. The editors explicitly state that we must describe this analysis as“intention to treat”in the checklist that they provided. Thus, we have modified our text from ‘modified intention to treat’ to ‘*Data analysis was conducted as modified intention to treat: participants were included in the complete case analysis if they had initiated at least one follow-up survey.*’ in the revised manuscript (see the first paragraph of the Statistical analysis). We hope that this is now clearer.

***Comment 9:*** P10, Analysis of Primary Outcome. Usually I would say Clopper-Pearson has undesirable qualities (i.e. conservative relative to more modern estimators) but with the sample size, and high prevalence of the outcomes it is safely applied here.

***Response:*** We agree with the reviewer that, unless n is quite large, the exact Clopper-Pearson interval can be overly conservative with coverage of at least (1-\alpha) instead of exactly (1-\alpha). We also know that the Wald interval can be too liberal in that it produces inadequate coverage when the sample size or p is too small. However, the sample sizes and proportions reasonable for all 95% CIs presented for binomial proportions. Thus, we modified these CIs to Wald CIs, and, as the reviewer pointed out, did not see much change. We appreciate this suggestion from the reviewer, as the p-values that we are required to present (per editor suggestion) will now coincide with the CIs since they are produced from a Wald test.

***Comment 10:*** P11. What about the comparison of SST vs SST + incentives at 3 months?

***Response:*** We have added this comparison to the main text (see the fourth paragraph of the Results section), *“Compared to the standard SST arm, there was no observed difference in the proportion of individuals testing for syphilis in the lottery incentivized SST arm during the first three months (RD: -0.3%, p=1.00, 95% CI: -12.2 to 11.8%).”*

***Comment 11:*** Table S2. This is a useful table - clearly the participants lost to follow-up were different on some of the key variables. When looking at the sensitivity analysis with imputation there are some good auxiliary variables that could be included based on this table.

***Response:*** We now include the following statement in Methods (see the second paragraph of the Statistical analysis),*“Compared to those who completed at least one follow-up survey, participants lost-to-follow-up differed on three demographic variables included in the logistic regression (Appendix p 11-12, Table S2): Annual income, disclosure as MSM to family, friends, or healthcare professionals, and ever tested for HIV.”*

***Comment 12:*** Table S4. This wasn't specified in the protocol - when was this sensitivity test planned? This type of imputation (similar to NRI) is an ok sensitivity test but the sensitivity test in the protocol (with MI) is more likely to give realistic results (assuming MAR).

***Response:*** Thank you, as the reviewer suggests, we have left the sensitivity tests for worst- and best-case scenario, but have added another sensitivity test with MI as follows (see the second paragraph of the Statistical analysis):

*“We also conducted sensitivity analyses on uptake of syphilis testing using non-responder imputation (NRI) to impute the worst- and best-case scenarios and multiple imputation (MI) by fully conditional specification (FCS) with logistic regression. For non-responder imputation, the sensitivity analyses involved replacing missing values with the worst value (not tested for syphilis) and best value (tested for syphilis) in the observed data. We assumed for the best value that all the men with missing follow-up data who applied for the syphilis self-testing kits received syphilis testing during the trial. For multiple imputation, Rubin’s rule was used to compute pooled estimates for the risk differences and standard errors. Compared to those who completed at least one follow-up survey, participants lost-to-follow-up differed on three demographic variables included in the logistic regression (Appendix p 6-7, Table S2): Annual income, disclosure as MSM to family, friends, or healthcare professionals, and ever tested for HIV.”*

**Response to reviewer #2**

***Comment :*** Figure 2. The box listing those exclusion states that '55 were currently living with syphilis.' This gives the impression that syphilis is like HIV regarding the lack of curable treatments. Consider using the same statement in the text (page 11 stating that 55 currently had syphilis).

***Response:***Thanks for the reviewer’s suggestion. We have rephrased the statement as ‘55 currently had syphilis’ in Figure 2 in the revised manuscript.

**Response to reviewer #3**

***Comment 1:*** However, the main concern is that the self-testing kits is dual syphilis/HIV self-testing kits, thus we do not know whether the increase of the proportion of the kits uptake is due to participants'interest in HIV testing or syphilis testing. The intervention is kind of a "dual intervention", while the authors only focused on part of the intervention and its result, which is not appropriate.

***Response:***Thanks for the reviewer’s comments. We understand the reviewer’s concern. Using dual testing kits may attract participants who were more interested in HIV testing results. In this study, we identified 18 men living with HIV, and eight men were newly identified through participating in this study. We did a sensitivity analysis by removing the 18 men from the study, and found little change to the original outcomes (see below). We have added a paragraph in the Discussion to clarify why we chose the Syphilis/HIV duo rapid test kits in the study (see the fifth paragraph of the Discussion). We also put it as a limitation to the study in the revised manuscript (see limitation in the Discussion section).

*Table Sensitivity analysis on the uptake of syphilis testing by removing 18 men infected with HIV*

|  | *Original outcome* | *Hypothesis outcome ** | *Original risk*  *(probability) difference^**^* | *Hypothesis risk (probability) difference^**^* |
| --- | --- | --- | --- | --- |
| *Standard of care* | | |  |  |
| *3-month* | *7/131(5.3)* | *7/131(5.3)* | *-* | *-* |
| *6-month* | *14/129(10.9)* | *14/129(10.9)* | *-* | *-* |
| *overall* | *20/136(14.7)* | *20/136(14.7)* | *-* | *-* |
| *Standard SST* | | |  |  |
| *3-month* | *74/137(54.0)* | *66/128(51.6)* | *48.7(39.5-57.9) ^#^* | *46.3(36.7-55.7) ^#^* |
| *6-month* | *51/139(36.7)* | *47/130(36.2)* | *25.8(16.2-35.5) ^#^* | *25.3(15.5-35.2) ^#^* |
| *overall* | *90/142(63.4)* | *81/133(60.9)* | *48.7(38.8-58.6) ^#^* | *46.2(36.0-56.4) ^#^* |
| *Lottery incentivized SST* | | |  |  |
| *3-month* | *72/134(53.7)* | *65/125(52.0)* | *48.4(39.1-57.7) ^#^* | *46.7(37.1-56.2) ^#^* |
| *6-month* | *69/126(54.8)* | *63/118(53.4)* | *43.9(33.7-54.1) ^#^* | *42.5(32.1-53.0) ^#^* |
| *overall* | *90/137(65.7)* | *81/128(63.3)* | *51.0(41.1-60.9) ^#^* | *48.6(38.3-58.8) ^#^* |

** Exclude participants infected with HIV, ^#^ P<0.05. ** compare to standard of care group*

*(Page 16) We used rapid dual self-testing for syphilis and HIV in this study, given the syndemic of syphilis and HIV among MSM globally.^38^ HIV self-testing has already created extensive infrastructure and public health pathways, including online testing programs, verification methods, integration of public health and community-based organization programs, and communication materials.^23,24^ Previous studies have shown the feasibility and necessity of integrating syphilis self-testing into HIV self-testing services.^4,24^ WHO also recommended using dual rapid tests for HIV and syphilis as the initial screening test in antenatal care.^39^ Our study suggests that integrating HIV and syphilis self-testing could contribute to the dual elimination of HIV and syphilis.^40^*

*(Page 18, limitation) Fifth, we used dual self-testing for syphilis and HIV in this study, which might raise the concern that the increase in the proportion of testing uptake is due to participants’ interested in the HIV testing. In this study, we identified 18 men living with HIV, and eight men were newly identified through participating in this study. We did a sensitivity analysis by removing the 18 men from the study, and found little change to the original outcomes.*

***Comment 2:*** In China, actually there is no free HIV and syphilis self-testing kits available. Thus, the SST arm provided free kits is a big offer for MSM, and could be recommended for public health policy makers, but why the authors design an SST +incentive arms? Since it needs more budget for government, and the implication is kind of far from the reality.

***Response:***Many MSM will not receive a free HIV/syphilis test even if the test kit is free. This suggests the need for generating demand for testing, including financial incentives.

***Comment 3:***The conclusion stated that :"…..particularly among men who had never tested for syphilis. I am thinking whether this conclusion is appropriate or not, since the enrollment is not stratified by non-testers or former testers.

***Response:***Thanks for the reviewer’s comments. We have removed this conclusion in the revised manuscript.

***Comment 3:*** The big difference between the two intervention groups with the standard of care group partly due to the social isolation during the COVID-19 outbreak during Jan to May 2020. Most of the places strongly recommended people to stay home, and some of the VCTs are closed, for example, those run by gay-friendly CBOs. Thus the last sentence on page 4 stated that facility-based testing is still available is not always the truth, since the participants were from 124 cities, and the lockdown measures might not be the same.

***Response:***We agree with the reviewer’s saying that most of the places strongly recommended people to stay home, and some of the VCTs are closed. We have provided a list of from where the participants were recruited, and of those, which cities were lockdown/clinic closure as an Appendix (Appendix p 25): the participants were enrolled from 124 cities in 26 provinces in China, and only three cities in Hubei province (Wuhan with 2 people, Xiantao with 2 people, Ezhou with 1 people ) were affected by lock downs/clinic closure during the recruitment period. However, even in those three cities, people could still get syphilis testing in the health facilities, but with a series of risk assessment procedures before they enter health facilities. We also have provided discussion on the impact of COVID-19 to our study in the sixth paragraph of the Discussion in the manuscript.

***Comment 4:*** Another concern is that, 54082 participants clicked the flyer link, and 52089 withdrew before consent, which means that most of the participants were not interested in this program. Thus the external validity of the study intervention need to be considered.

***Response:***Thanks for the reviewer’s comments. The potentials reasons of many participants withdrawing before consent include: 1) Repeat clicking of the link. Our survey was restricted to one phone number and a single device (IP address). After the survey was completed, the phone number and IP address connected to that survey could not be used to access the survey again. However, if participants were unavailable to complete the survey in one sitting, they would need to reenter the survey link several times in order to finish the entire survey. Additionally, some participants who finished the survey may attempt to complete the survey again to obtain a subsidy, although the system would tell them they could only participate one time. 2) we recruited participants from a large MSM-oriented mobile social app - Blued (Danlan, Beijing, China). We designed a vivid invitation poster which was posted on Blued’s portal website and startup screen. The poster would jump out and last for four seconds when people entered into the website to inform them that they would have a chance to get free syphilis self-testing kits if they participated in the study. There were many people who might be interested in getting free SST kits, but withdrew after clicking the poster (link) when they discovered that there was a survey requirement. Additionally, there might be people who just clicked the poster unintentionally. By February 2018, Blued had 40 million registered users globally, with 70% in Mainland China. Therefore, the number in those two cases above would not be small. We have also discussed the generalizability issue in the third paragraph of the Discussion section in the manuscript.

***Comment 5:*** Among the 2124 men who are interested in this study, 1362 reported no condomless anal sex during the previous year, which is much different from the general MSM population.

***Response:***Thanks for the reviewer’s comments. We made a search in PubMed and found that most studies examined the outcome within the past six or three months, not in the past year. Limited studies^a,b^ examining the outcome in the past year reported a percentage of no condomless anal sex ranging from 36.5% to 60.0%, which is a bit lower than our results (64.1%).

Reference

a. Zhang J, Xu J, Leuba S I, et al. Increasing Condomless Anal Intercourse and HIV Prevalence Among Men Who Have Sex with Men Who Have Never Been Tested for HIV Before: A Serial Cross-Sectional Study in Shenyang, China from 2012 to 2016[J]. AIDS and Behavior, 2020, 24(10): 2918-2926.
b. Sun S, Hoyt W T, Pachankis J E. Sexual risk behaviors in the internet age: the case of Chinese men who have sex with men[J]. AIDS care, 2020, 32(3): 302-309.

***Comment 6:*** On page 5, the cost of the kit has a range, which means on market the kit has range of cost, or in this study? Typically the cost of the kit in this study should be same.

***Response:***That range refers to range of cost on market, not in this study. The cost of the kits used in this study were the same.

***Comment 7:*** What do you mean by "online informed consent"? do they provided written informed consent?

***Response:*** We have specified the process of obtaining the informed consent in the revised manuscript (see the sixth paragraph of Methods section).

*All participants provided online informed consent before the baseline survey by clicking on a “button” indicating that the participant has read the consent form and agrees to participate.*

***Comment 8:*** How many of kits has been delivered to the participants per month (and in total)? And how much proportion of those kits were tested for themselves, and how much for partners or others?

***Response:*** We delivered 523 kits to the participants in total, of which, 504 (96.4%) tested for themselves and 19 (3.6%) tested for partners or others. We provided a list for the details below.

| **Month** | **No. of delivered kits** | **No. of kits tested for themselves (%)** | **No. of kits tested for partners or others (%)** |
| --- | --- | --- | --- |
| The 1st | 167 | 167 (100) | 0 |
| The 2nd | 65 | 59 (90.7) | 6 (9.3) |
| The 3rd | 18 | 14 (77.8) | 4 (22.2) |
| The 4th | 139 | 139 (100) | 0 (0) |
| The 5th | 112 | 107 (95.5) | 5 (4.5) |
| The 6th | 22 | 18 (81.8) | 4 (18.2) |
| Total | 523 | 504 (96.4) | 19 (3.6) |
