# Supplementary material for: Expanding syphilis test uptake using rapid dual self-testing for syphilis and HIV among men who have sex with men in China: A multiarm randomized controlled trial
Source: PLoS Med. 2022 Mar 2;19(3):e1003930. doi: 10.1371/journal.pmed.1003930 (PMC8890628; doi:10.1371/journal.pmed.1003930)
Supplement: Attachment — Submitted filename: Response to Editor and Reviewer-29Dec2021.docx [file pmed.1003930.s015.docx]

Dear PLOS Medicine Editors

Thank you for the opportunity to respond to the helpful reviewer comments. Find attached a tracked changes and a clean version of the manuscript. Below are point-by-point responses and italicized text refers to the new text within the manuscript.

Thank you for your consideration.

Sincerely,

Wang Cheng, Ann Marie Weideman, and Joe Tucker on behalf of the co-authors

**Responses to the editors**

***Comment 1:***  With regard to data access, do you have contact information (web or email address) for a local authority in China that can grant permission?

***Response:*** Yes, we have revised the Data availability statement as below: De-identified data cannot be made open accessed because of the IRB decision to ensure patient confidentiality. Permission can be requested by contacting the Guangdong Center for Skin Diseases and STI Control, China (contact via gdpfzx@vip.163.com).

***Comment 2:***  Please add line numbers in the next revision.

***Response:*** We have added line numbers in the revised manuscript.

***Comment 3:*** Author summary
a) Under “Why was this study done?”, 2nd line should read “….expand syphilis testing”.
b) adapting the author summary to present the findings in a narrative way rather than repeating all the numbers already in the abstract.

***Response:*** a) We apologize for the typo. We have revised the text accordingly. b) We have revised the author summary accordingly in the revised manuscript (see below) .

## *What Did the Researchers Do and Find?*

- *We recruited 451 MSM participants in a three-arm randomized controlled trial from 124 cities in 26 Chinese provinces between 7 January 2020 and 17 July 2020.*
- *Our study showed that promoting syphilis self-testing among MSM substantially increased syphilis test uptake compared with the standard-of-care.*
- *The cost per person tested is cheaper for the syphilis self-testing arm compared to the standard-of-care arm.*

***Comment 4:*** In the abstract and main text, please rephrase “win $15 if they tested for syphilis” to “…win $15 if they had a syphilis test or similar…”.

***Response:*** We have rephrased “win $15 if they tested for syphilis” to “win $15 if they had a syphilis test” in the revised manuscript.

***Comment 5:*** Please add p values (including “p<0.001”) after the CIs.

***Response:*** We have added p values (including “p<0.001”) after the CIs in the revised manuscript.

***Comment 6:***  Please do not report P<0.0001; report as P < 0.001.

***Response:*** We have changed the P<0.0001 to P<0.001 in the revised manuscript.

**Response to the Academic editor**

***Comment 1:***  I continue to have doubts about generalizability. I would like to see a paragraph in the discussion that addresses the generalizability of results to the entire potentially eligible population and makes clear that the sampling strategy, very large number of self-excluders, potential for disclosure, etc. all limit the applicability of results to a subset of the MSM population who are willing to be identified as wanting a syphilis/HIV test. As it stands, there is an implication that the findings would be relevant for the entire population, which is not correct.

***Response:*** We agree that the generalizability statement could be more clear (see below, the fourth paragraph of the Discussion). However, in terms of identification and privacy risk, there were several ways to decrease “outing” and a systematic review found that self-testing was associated with decreased stigma compared to facility-based testing approaches.[a] Men were not required to provide any identifying information, including real names, IP addresses, and physical home addresses.

*Our study findings may be generalizable to the many other settings where MSM seek online public health information that can be accessed without providing identifying information. Online platforms represent a major opportunity for reaching marginalized MSM and designing pragmatic interventions.[40] Online platforms can reach MSM in remote rural areas, MSM who do not tell doctors about their same-sex behaviors (an especially important consideration in low and middle-income countries with severe stigma), and other vulnerable subsets of MSM. At the same time, the approach used in this study requires an online connection, willingness to use an online platform for requesting testing, and a postal service to mail the kits. Our empirical generalizability research found that MSM population characteristics from an online MSM trial were comparable to data from a national, cross-sectional sample of MSM [41].*

Reference:

a.Yilu Q I N, Larry H A N, Andrew BABBITT J S W, et al. Experiences using and organizing HIV self-testing: a global qualitative systematic review[J]. AIDS (London, England), 2018, 32(3): 371.

**Response to reviewer #1**

***Comment 1:*** The extra information about recruitment (from the splash screen of the app) explains the drop-out rate. From my involvement with other work with similar approaches to recruitment via an app (although with a different target population) there was low 'click-through' rate to consent to recruitment. There probably isn't an easy way around this, the views of the subject-matter reviewers familiar with the population are important here in establishing if generalisability is likely to be an issue.

***Response:*** We agree that inferences from this data should be made with caution. We have revised the paragraph on generalizability to underline this point.

***Comment 2:*** The sensitivity analyses around the missing data are reassuring. For the MI sensitivity analysis some additional detail is needed in the methods (e.g. number of imputations, rationale for the number of imputations chose, what variables were used in the imputation model). Table S4 (or another table) should include a repeat of the main analysis with the imputed datasets. Given the summary data it's very unlikely the conclusions would be different from the main analysis but it should still be included.

***Response:***

We agree that it would be helpful to include additional details regarding the MI methods in the text. We have amended Methods to include the following:

*For multiple imputation, Rubin’s rule[27] was used to compute pooled estimates for the risk differences and standard errors. The number of imputations was selected based on relative efficiency such that* ${FMI_{max}}/m\approx0.01$*, where* $FMI_{max}$ *is the maximum fraction of missing information (computed as in Raghunathan, 2015) and* $m$ *is the number of imputations. The FMI ranged from 0.02 to 0.21, so* $m=20$ *was chosen for the number of imputations.* *Compared to those who completed at least one follow-up survey, participants lost-to-follow-up differed on three demographic variables included in the imputation model (Appendix p 6-7, Table S2): Annual income, disclosure as MSM to family, friends, or healthcare professionals, and ever tested for HIV.*

We also agree that Table S4 should include a repeat of the main analysis (uptake of syphilis testing) with the imputed datasets. This was included in the last version of the Appendix as a final column in Table S4. This column is labeled “Original vs MI” and summarizes the estimated risk differences and corresponding CIs for standard-of-care, standard SST, and lottery incentivized SST. There were no observed differences between the original analysis and the analysis performed using MI.

**Response to reviewer #3**

***Comment 1:*** In the abstract: The conclusion of "Compared to standard-of-care, providing SST significantly increased the proportion of MSM testing for syphilis in China and was cheaper (per person tested)."---- Since the "SST is short for "syphilis self testing", then I think "providing SST" should be specified as "providing SST plus HIV self-testing dual kits", since the study is not examining the effectiveness of providing single SST kits. And the same problem during the Author Summary and other parts of the main text.

***Response:*** Thanks for the reviewer’s suggestion. We have explicitly stated that we used rapid dual self-testing for syphilis and HIV in the Title and Methods section. In the Conclusion section and Author Summary, the SST is more likely to be referred to SST strategy. We have conducted a sensitive analysis to examine the effect of using dual self-test kits on the primary outcome (see the seventh paragraph of the Discussion).

*Fifth, we used dual self-testing for syphilis and HIV in this study, which might raise the concern that the increase in the proportion of testing uptake is due to participants interested in the HIV testing. In this study, we identified 18 men living with HIV, and eight men were newly identified through participation in this study. We conducted a sensitivity analysis by removing the 18 men from the study, and found little change to the original outcomes (results not shown).*

***Comment 2:*** On page 16: "Previous studies have shown the feasibility and necessity of integrating syphilis self testing into HIV self-testing services.4,24 WHO also recommended using dual rapid tests for HIV and syphilis as the initial screening test in antenatal care." --------With this information already in the literature, the authors should justify the significance of this study, since WHO has recommended and China also conducted in reality (many CDC and Community-based organizations used duo testing kits in daily practice).

***Response:*** WHO only recommended using dual rapid tests for HIV and syphilis in antenatal care. In many low- and middle- income countries, syphilis is still a major public health problem, but the syphilis testing uptake remains low, especially among MSM. In most countries, syphilis testing occurs in health facilities, but existing facility-based syphilis testing and management resources and services are inadequate to curb the spread of syphilis. This study is the first randomized controlled trial to evaluate the effectiveness and cost of syphilis self-testing, extending the limited literature on syphilis self-testing. Our randomized controlled trial and economic evaluation strengthens the evidence for the value of implementing syphilis self-testing programs among MSM. More justification and implication can be found in the Background (see the 1^st^ and 2^nd^ paragraph) and Discussion section (see the 6^th^ paragraph).

***Comment 3:*** What so called the "online informed consent" is more like an information notification rather than informed consent. Usually IRB requires written or oral IC.

***Response:*** While we agree that non-digital research typically requires written or oral informed consent, there are increasing digital studies that use online informed consent. There is an empirical literature suggesting that online consent is similar to written consent in terms of participant comprehension.[a]

Reference:

a.Varnhagen CK, Gushta M, Daniels J, Peters TC, Parmar N, Law D, Hirsch R, Takach BS, Johnson T. How informed is online informed consent? Ethics Behav. 2005;15(1):37-48.

***Comment 4:*** The inclusion criteria for having condomless anal sex is previous 12 months, but having no syphilis test is previous 6 months. Why the time point selection is different? What is the rational? [as stated by authors, in literature, usually they evaluated condomless sex in recent 6 months, 3 months and the last time.

***Response:*** Sexually active MSM were recommended to be tested at least annually for syphilis, with testing every 3-6 months for MSM at elevated risk.[1,2] Men who had condomless anal sex in the past 12 months with no syphilis test in the past 6 months were thought to be linked to an increased risk of syphilis transmission, and should be paid attention.[3-5] Additionally, from the implementation perspective, it’s difficult to recruit men who had condomless anal sex in previous six months, especially in the context of COVID-19 pandemic.

Reference

1. Aberg JA, Gallant JE, Ghanem KG, Emmanuel P, Zingman BS, Horberg MA; Infectious Diseases Society of America. Primary care guidelines for the management of persons infected with HIV: 2013 update by the HIV medicine association of the Infectious Diseases Society of America. Clin Infect Dis. 2014 Jan;58(1):e1-34. doi: 10.1093/cid/cit665. Epub 2013 Nov 13. PMID: 24235263.
2. Workowski KA, Bolan GA; Centers for Disease Control and Prevention. Sexually transmitted diseases treatment guidelines, 2015. MMWR Recomm Rep. 2015 Jun 5;64(RR-03):1-137. Erratum in: MMWR Recomm Rep. 2015 Aug 28;64(33):924. PMID: 26042815; PMCID: PMC5885289.
3. Barron-Limon S, Semple SJ, Strathdee SA, Lozada R, Vargas-Ojeda A, Patterson TL: Correlates of unprotected anal sex among men who have sex with men in Tijuana, Mexico. BMC PUBLIC HEALTH 2012, 12:433.
4. Hongbo Jiang , Xiaobin Chen , Jing Li , Zhimin Tan , Weibin Cheng , Yi Yang.Predictors of condom use behavior among men who have sex with men in China using a modified information-motivation-behavioral skills (IMB) model. BMC Public Health . 2019 Mar 4;19(1):261.
5. Kramer SC, Schmidt AJ, Berg RC, Furegato M, Hospers H, Folch C, Marcus U: Factors associated with unprotected anal sex with multiple non-steady partners in the past 12 months: results from the European Men-Who-Have-Sex-With-Men Internet Survey (EMIS 2010). BMC PUBLIC HEALTH 2016, 16:47
