# Supplementary material for: Expanding syphilis test uptake using rapid dual self-testing for syphilis and HIV among men who have sex with men in China: A multiarm randomized controlled trial
Source: PLoS Med. 2022 Mar 2;19(3):e1003930. doi: 10.1371/journal.pmed.1003930 (PMC8890628; doi:10.1371/journal.pmed.1003930)
Supplement: Attachment — Submitted filename: Response to Editor and Reviewer-22Jan2022.docx [file pmed.1003930.s016.docx]

**Responses to the editors**

***Comment 1:***  Title: Please remove“in China” from the subtitle and place it right before the colon. It should read“Expanding syphilis test uptake using rapid dual self-testing for syphilis and HIV among men who have sex with men in China: a multi-arm randomized controlled trial”.

***Response:*** We have removed “in China” from the subtitle and place it right before the colon in the revised manuscript.

***Comment 2:***  Abstract: Please remove the following sentence from the end of the Methods and Findings section: “The trial is registered with the Chinese Clinical Trial Registry, number ChiCTR1900022409. The study design and results were reported according to CONSORT 2010 guidelines.”

***Response:*** We have removed the sentence from the Methods and Findings section in the revised manuscript.

***Comment 3:*** Please replace“Chinese MSM”with "MSM in China."
***Response:*** We have replaced “Chinese MSM” with “MSM in China” throughout the text in the revised manuscript.

***Comment 4:*** Please ensure that journal name abbreviations consistently match those found in the National Center for Biotechnology Information (NCBI) databases. [https://journals.plos.org/plosmedicine/s/submission-guidelines#loc-references](https://journals.plos.org/plosmedicine/s/submission-guidelines#loc-references). For example, PLOS Medicine should be PLOS Med.

***Response:*** We have checked and revised all the jounal name abbreviations again, and ensure that they have matched those found in the NCBI.
